# The genomics of adaptation to climate in European great tit (*Parus major*) populations

**DOI:** 10.1093/evlett/qrad043

**Published:** 2023-10-12

**Authors:** Joanne C Stonehouse, Lewis G Spurgin, Veronika N Laine, Mirte Bosse, The Great Tit HapMap Consortium, Martien A M Groenen, Kees van Oers, Ben C Sheldon, Marcel E Visser, Jon Slate

**Affiliations:** School of Biosciences, University of Sheffield, Sheffield, United Kingdom; School of Biological Sciences, University of East Anglia, Norwich Research Park, Norwich, United Kingdom; Department of Animal Ecology, Netherlands Institute of Ecology (NIOO-KNAW), Wageningen, The Netherlands; Finnish Museum of Natural History, University of Helsinki, Helsinki, Finland; Animal Breeding and Genomics, Wageningen University & Research, Wageningen, The Netherlands; Amsterdam Institute for Life and Environment (A-LIFE), Section Ecology and Evolution, Vrije Universiteit Amsterdam, Amsterdam, The Netherlands; Animal Breeding and Genomics, Wageningen University & Research, Wageningen, The Netherlands; Department of Animal Ecology, Netherlands Institute of Ecology (NIOO-KNAW), Wageningen, The Netherlands; Edward Grey Institute, Department of Biology, University of Oxford, Oxford, United Kingdom; Department of Animal Ecology, Netherlands Institute of Ecology (NIOO-KNAW), Wageningen, The Netherlands; School of Biosciences, University of Sheffield, Sheffield, United Kingdom

**Keywords:** climate adaptation, great tit HapMap project, signatures of selection, genome-environment association (GEA), GO term enrichment, climate change

## Abstract

The recognition that climate change is occurring at an unprecedented rate means that there is increased urgency in understanding how organisms can adapt to a changing environment. Wild great tit (*Parus major*) populations represent an attractive ecological model system to understand the genomics of climate adaptation. They are widely distributed across Eurasia and they have been documented to respond to climate change. We performed a Bayesian genome-environment analysis, by combining local climate data with single nucleotide polymorphisms genotype data from 20 European populations (broadly spanning the species’ continental range). We found 36 genes putatively linked to adaptation to climate. Following an enrichment analysis of biological process Gene Ontology (GO) terms, we identified over-represented terms and pathways among the candidate genes. Because many different genes and GO terms are associated with climate variables, it seems likely that climate adaptation is polygenic and genetically complex. Our findings also suggest that geographical climate adaptation has been occurring since great tits left their Southern European refugia at the end of the last ice age. Finally, we show that substantial climate-associated genetic variation remains, which will be essential for adaptation to future changes.

## Teaser

Whether and how organisms adapt to variations in climate remains a challenging question. However, advances in ecological genomics tools and statistical genetics methods have made it feasible to identify genomic regions that have possibly responded to selection. Here we study pan-European populations of the ecological model species, the great tit (*Parus major* ), to try to identify genes and genetic pathways that have possibly enabled different populations to adapt to past and present climate conditions. By: (i) genotyping 20 different great tit populations at 479,590 single nucleotide polymorphisms, and looking for associations with climate data collected from each location between the 1970s and 2000s, and (ii) using Gene Ontology enrichment analysis to infer the biological roles of climate-associated genes, we aimed to better understand the genetics of climate adaptation. Up to 36 different genes were found to be significantly associated with adaptation to climate, and several different biological processes were over-represented among these genes. Our findings suggest that adaptation to multiple climate variables has occurred, with many genes enabling different populations to adapt to their current and past environmental climatic conditions. It is likely that adaptive genetic variation persists in contemporary great tit populations.

## Introduction

Terrestrial ecosystems face an unprecedented rate of climate change in the coming decades. A 1.5°C global surface temperature warming (above pre-industrial levels), caused by human activities and emissions of greenhouse gases and aerosol pathways, is likely by 2040 (IPCC, 2022). Likely impacts on climate include an increase in the number of regions with raised hot and cold temperature extremes per season and changes to the intensity, frequency, and duration of extreme weather events, such as heatwaves, drought, and flooding (Eyring et al., 2016). Further consequences of climate change that threaten populations include forest fires, extreme weather events, the spread of invasive species, pests and disease, and the decreased functioning of ecosystem services. Hence, an urgent issue is understanding how organisms respond to the changing environment (IPCC, 2022; Root et al., 2003). How a species’ physiology and behavior tolerate changes to the local environment will determine its fitness and survival, and therefore the need to respond to climate change (Cheviron & Brumfield, 2012; Khaliq et al., 2014). Sessile plants, low-dispersal insects, and some unique or threatened vertebrates, e.g., small island populations, are especially vulnerable to local extinction if their response is insufficient (Khaliq et al., 2014; Parmesan & Yohe, 2003), although a failure to adapt to climate change is expected to be consequential in any population.

Where populations are able to respond to climate change, they can do so in three possible ways: (1) Populations may shift range to more suitable conditions. For example, poleward distribution shifts in response to anthropogenic climate warming are observed in many plants, birds, and butterflies (Chen et al., 2011; Devictor et al., 2012; Hickling et al., 2006; Parmesan et al., 1999; Sunday et al., 2012). (2) Through plasticity of key phenotypic traits, such as behavior and the timing (phenology) of reproduction and migration (Parmesan, 2006). (3) By evolving to the new conditions, i.e., adaptation via selection favoring particular alleles that have some form of fitness advantage. However, the evolution of climate adaptation due to specific genomic regions/genes might not be fast enough to match climate change, especially if multiple climatic variables are changing simultaneously and/or generation times are long. The evolutionary potential to adapt has consequences for population persistence (Khaliq et al., 2014; Parmesan, 2006). Furthermore, options (2) and (3) are not necessarily mutually exclusive, as both processes can occur, and plasticity itself may be heritable (i.e., an evolvable trait). Understanding the role of plastic vs. microevolutionary changes in response to climate change requires further investigation in many taxa, including birds (Charmantier & Gienapp, 2014), the focus of this study. Here we explore associations between genetic variation putatively under selection and climatic variables, in order to better understand microevolutionary responses to climate.

There are two distinct approaches for using genomic data to identify adaptive genetic variation and signatures of natural selection. First, molecular quantitative genetics approaches using phenotypic information. For example, linkage mapping or genome-wide association studies can be used to identify quantitative trait loci linked to phenotypes involved in adaptation (Santure & Garant, 2018). Where phenotypic data are lacking, a second approach, population genomics, uses genomic information (but no phenotypic measurement) to identify signatures of adaptive genetic variation and then attempts to relate genome regions under selection to evolutionary processes and environmental variation (Rellstab et al., 2015). Population genomics attempts to find regions of the genome that are more highly differentiated between populations than is expected from neutral processes—i.e., to find “outlier loci,” that are inferred to be involved in local adaptation (Butlin, 2010; Luikart et al., 2003). Outlier locus approaches have been used extensively to reveal signatures of local adaptation, e.g., to different host plants in walking-stick insects (Nosil et al., 2012), to different shoreline environments in marine snails (Butlin, 2010), and to different predator regimes in three-spine sticklebacks (Mazzarella et al., 2016). In birds, outlier loci associated with adaptation to living at high altitudes in Tibetan ground tits (Qu et al., 2013), to different forest habitats in blue tits (Perrier et al., 2020), and to urban landscapes in great tits (Salmón et al., 2021) have been found. An extension of the outlier locus approach is to use genome-environment association (GEA) methods to identify loci whose divergence is correlated with an environmental covariate of interest. GEA analyses are more challenging to implement than outlier loci analyses because they require: (i) multiple populations, (ii) a measure of the environmental variable of interest, and (iii) confidence that the environmental variable is the agent of selection. For comparisons of genome-scan methods, see Gautier (2015), Forester et al. (2018), and Booker et al. (2023).

Terrestrial species with populations that occupy a wide diversity of habitats/climatic conditions, but where gene flow persists, provide ideal systems to investigate the genomic responses to past (e.g., since the last ice age 10,000–15,000 years ago) and more recent (e.g., post-industrialization) climate changes (Rellstab et al., 2015). Ongoing gene flow is useful because the resulting relatively low differentiation across the genome makes regions responsible for adaptation more likely to stand out as outliers (Luikart et al., 2003). The aim of this article is to look for evidence across the genome of climate adaptation in a well-studied, model wild vertebrate species, the great tit *Parus major*. Previous genome-wide “outlier locus” analyses in great tits have not considered whether allele frequencies covary with environmental variables (Bosse et al., 2017; Spurgin et al., 2019), and so the agents of selection could not be identified.

The great tit is a well-suited species to understanding the genomics of climate adaptation because: (i) they occupy a wide range of environments and climates across Eurasia, with range edges extending into Eastern Russia, Fenno-Scandinavia, and North Africa (Gosler et al., 2020). (ii) As cavity breeders they readily use nest boxes, which has allowed long-term monitoring of wild populations, e.g., in Wytham Woods, UK (Lack, 1964) and Hoge Veluwe, NL (Van Balen, 1973). (iii) They are generally non-migratory, although some migration is observed in northern populations (Kvist et al., 1999), so populations should be well adapted to their local environment. Dispersal distances are usually short, but long-distance dispersal of some individuals facilitates gene flow among subpopulations, meaning there is only minor population differentiation, with *F*_ST_ typically around 0.01 (Laine et al., 2016; Lemoine et al., 2016; Spurgin et al., 2019; van Bers et al., 2012). The current distribution has likely spread after the last ice age from a single restricted refugium in southeastern Europe (Spurgin et al., 2019).

Great tits are sensitive to climate change, with warmer spring temperatures causing earlier egg-laying across Europe (Charmantier et al., 2008; Cole et al., 2021; Visser et al., 1998, 2021). The timing of breeding is especially important in the tri-trophic food web (oak bud burst-winter moth caterpillar-great tit), to ensure peak food resources to raise chicks (Burgess et al., 2018; Hinks et al., 2015; Perrins, 1970; Visser et al., 1998). An optimal phenology is critical for population persistence (Vedder et al., 2013 but see Reed et al., 2013). Great tits, and passerines more generally, may have sufficient phenotypic plasticity in egg lay date to track environmental changes, enabling them to cope with recent rapid rates of spring warming in the U.K. (Phillimore et al., 2016), although this plastic response is complex (Ramakers et al., 2018). If limits to phenotypic plasticity prevent an ongoing response to global climate change across parts of the distribution of *Parus major*, then evolutionary changes will be necessary to enable future population resilience and survival.

Rich genomic resources are available for genetic research of great tits. It was only the second passerine to have a high-quality reference genome sequenced and annotated (Laine et al., 2016); a genetic linkage map has been made (van Oers et al., 2014); DNA samples are available from multiple populations; a high-density ~500K single nucleotide polymorphism (SNP) chip has been produced (Kim et al., 2018); and the SNP chip has been used to genotype individuals from approximately 40 wild populations (Kim et al., 2018; Salmón et al., 2021; Spurgin et al., 2019). Samples for the cross-population analyses were contributed by the Great Tit HapMap Consortium, a group of researchers conducting field studies of great tits across much of the species’ range. The consortium was established with the aim of characterizing interpopulation genetic variation and hopefully identifying genes associated with local adaptation. Great tit populations have low *F*_ST_ across the genome, which means any outlier loci should stand out against a low background of differentiation. In addition, regions of the genome under natural selection have already been identified using outlier locus approaches (Bosse et al., 2017). Responses to selection appear to be greatest in populations at the range edges and are perhaps associated with morphology, plumage color, and stress response (Spurgin et al., 2019).

Here we address the question: What regions of the genome have enabled a wild bird species to adapt to different climate conditions? We first describe a genome-environment analysis (GEA) to identify genes that are associated with climate adaptation and show signatures of selection. We note, however, that an association with climate is not definitive evidence of adaptation to climate. Next, we use a Gene Ontology (GO) term enrichment analysis to identify over-represented biological functions among the genes with climate-associated SNPs.

## Materials and methods

### The great tit HapMap project field data and genotyping

Members of the great tit HapMap project consortium (Spurgin et al., 2019) provided blood samples from wild European great tit populations that are the focus of ongoing ecological studies. A total of 838 great tits from 29 locations in Europe were sampled across 22 countries, encompassing much of the species range. In most cases, the DNA was extracted using ammonium acetate/high salt extraction protocols (Kim et al., 2018), although a small number of samples were sent as extracted DNA. A custom-developed Affymetrix® great tit 650K SNP chip (Kim et al., 2018) was used to genotype the samples at Edinburgh Genomics, UK.

### Data filtering and QC

**Figure 1.**
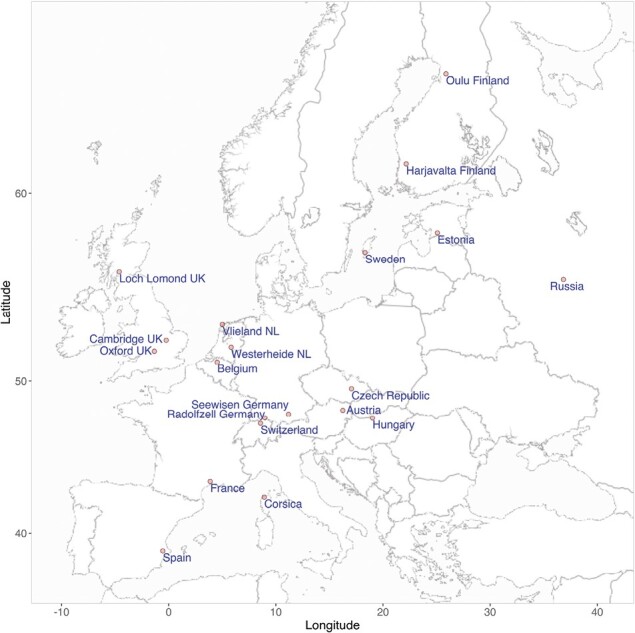
The 20 great tit population locations. Further details of each population are provided in Supplementary Table S1. Map tiles by Stamen Design (https://stamen.com), under CC BY 3.0. Contains data by OpenStreetMap (http://openstreetmap.org/copyright), under ODbL.

Following filtering approaches described elsewhere (Bosse et al., 2017; Spurgin et al., 2019), the data set contained a total of 647 great tits from 29 populations, typed at 483,888 SNPs. Filtering was applied in PLINK v1.90 (Chang et al., 2015) with parameter thresholds: --maf (minor allele frequency) 0.01, --geno (proportion of individuals typed at an SNP) 0.8, --not-chr 35 (remove markers from “chromosome” 35), and --rel-cutoff (relatedness cutoff) 0.25. This removed SNPs that were almost lacking in variation, that had high rates of missing data, or that were assigned to an unknown chromosome (chr 35 is a collection of scaffolds on unknown chromosomes in our Plink files). Only males were included for the Z chromosome, as females are hemizygous. In addition, populations with less than 15 individuals were excluded. The HapMap dataset is known to include some samples of close relatives, such as trios of parents and chicks. Having close relatives causes much higher amounts of population structure, which can give misleading results in population genetic analyses. Pairwise-relatedness was estimated in PLINK and the dataset filtered, to exclude one member of a pair when the relatedness was greater than 0.25. This step ensured only one individual per trio was used. After passing quality control, 479,590 SNPs and 535 samples were retained from 20 populations across Europe (Figure 1; Supplementary Table S1).

### Climate data collection and principal component analysis

The WorldClim v2 data set provides good spatial resolution climate data across Europe (interpolated from monthly averaged weather station records 1970–2000) (Fick & Hijmans, 2017). Twenty-two variables (19 bioclimate variables, solar radiation, wind speed, and water vapor pressure) were extracted for the 20 population locations (Supplementary Table S2). The complexity of the dataset was reduced by a principal component analysis (PCA), using the FactoMineR package v2.4 (Lê et al., 2008), run in R version 3.6.1. Downstream analyses used the first four principal components (PCs) for each location, scaled to mean = 0, *SD* = 1. Temperature and precipitation have been demonstrated to be associated with constraints on great tit growth rate (Eeva et al., 2020; Rodriguez & Barba, 2016). More generally, a review of climatic effects on nestling growth and development in birds (Sauve et al., 2021) has highlighted solar radiation, wind speed, and water vapor pressure as additional variables that might affect juvenile traits. Clearly, early life development is sensitive to climate effects (via, for example, changes in food availability and parental care), with potentially long-lasting effects on adult phenotypes and fitness. Thus, we decided to use all 22 available climatic variables, rather than *a priori* choose some and reject others.

### Genome-environment analysis: Detecting loci potentially involved in adaptation to climate

To identify climate-associated SNPs, we used BayPass 2.1 (Gautier, 2015), a software package that has been used to identify climate-associated loci in similar studies of *Arabidopsis thaliana* (Frachon et al., 2018), barley (Contreras-Moreira et al., 2019), and Mediterranean cattle breeds (Flori et al., 2019). We elected to use BayPass over redundancy analysis as it controls for population structure, can handle missing genotype data, and is regarded as quite conservative in declaring an SNP as an outlier (Forester et al., 2018). We used BayPass in a two-step process, using default parameters unless stated otherwise. First, we ran the core model to calculate XtX, a differentiation statistic for each SNP. The core model also estimates the covariance in allele frequencies (in the form of a matrix Ω) that arises as a result of population structure; thus, demographic effects that can otherwise cause false positives are accounted for. For further details of XtX, see Coop et al. (2010) and Günther & Coop (2013). The significance of the observed XtX statistics was obtained by generating a null XtX distribution from pseudo-observed data (POD) simulated with 400K SNPs. Variants identified as highly differentiated “outlier” SNPs at a 1% significance threshold were regarded as being loci putatively under selection. Note that at this stage, no associations with climatic variables have been tested for.

Second, for all 479,520 SNPs, the default auxiliary (AUX) model with scaled covariance matrix and climate covariates was run independently three times (with different starting seeds) for the four PCs (analyzed jointly in each run). Associations between each SNP and each climate PC were judged from Bayes factors (expressed in deciban units, dB) under an Markov Chain Monte Carlo (MCMC) sampling method (*BFmc*); evidence for a SNP being associated with climate adaptation was judged by Jeffreys’ rule (Jeffreys, 1961): 10<BFmc≤
15 “strong evidence”; 15<BFmc≤
20 “very strong evidence”; and *BFmc* > 20 “decisive evidence.” The evidence was evaluated by comparing the observed associations with a null distribution generated from POD simulated using the Ω generated in the core model, i.e., to realistically simulate the true demographic history. A variant was evaluated on the strength of evidence threshold across all three runs, i.e., a variant would only be considered as providing “decisive” evidence for association with a climate variable if it scored >20 for all three runs. The best potential candidate loci for climate adaptation were classified as SNPs that were identified as being both associated with climate (from the AUX analysis) and as an “outlier” variant under selection (from the core analysis).

### Annotation of candidate loci for climate adaptation

All SNPs were annotated using the great tit reference genome (GCF_001522545.3_Parus_major1.1_genome.gff) converted to bed file with bedops (Neph et al., 2012), with the closest gene identified using bedtools closest sub-command (Quinlan, 2014). This process annotated the gene the SNP was located in or the nearest gene within 2.5 kbp in either direction from the SNP. SNPs were regarded as unannotated if no gene was found within 2.5 kbp. An annotation window of 5 kb was chosen because in great tits linkage disequilibrium falls to almost baseline levels within 10 kbp or less (Spurgin et al., 2019). Of the 479,590 variants considered, 278,861 variants were annotated and assigned to 14,529 unique genes (i.e., most genes have more than one annotated SNP).

### Gene Ontology (GO) term enrichment analysis

#### Testing for GO term enrichment among potential climate adaptation loci

A GO term (Ashburner et al., 2000; The Gene Ontology Consortium, 2021) analysis on gene scores was performed (separately for each of PC1–PC4), to look for GO terms enriched among the genes with climate-associated SNPs against the background annotated gene universe for all of the SNPs on the chip. For the 14,529 genes containing SNPs, or with SNPs within 2.5 kbp, 8,867 great tit GO terms were retrieved from Ensembl (Ensembl Genes 109, *Parus major_1.1*), using the biomaRt package v3.8 (Durinck et al., 2005, 2009). To avoid over-rewarding genes with many SNPs, we took an approach similar to one described elsewhere (Dickson et al., 2020). For each gene, the number of SNPs in the gene or within 2.5 kbp of it was counted. Next, the maximum BayPass BF statistic of any SNP in that gene was determined. Then, all genes with that number of SNPs were ranked by max(BF), and a “*p* value” was determined by dividing the rank by the total number of genes with that number of SNPs. For example, if there were 100 genes with eight SNPs, then the gene with the greatest max(BF) was given a value of 1/100 = 0.01, the gene with the second greatest max(BF) was given a value of 2/100 = 0.02, etc. Relatively few genes had a very large number of SNPs (427 genes had 100 or more SNPs), so binning was performed for genes with 100–149 SNPs, 150–199 SNPs, 200–299 SNPs, 300–499 SNPs, and ≥ 500 SNPs. In total, 14,529 genes were included in the analysis. Note that previous approaches calculated the mean test statistic across all SNPs within a gene (Dickson, 2020), rather than using the maximum value observed at any SNP. Here, the maximum is appropriate, because linkage disequilibrium declines rapidly in great tits, meaning even true outlier loci are not expected to have a signal that extends across the gene.

Significance testing for GO term enrichment was performed with the topGO v2.36.0 (Alexa & Rahnenführer, 2020) Bioconductor (Release 3.9) package in R. The data container used as input for topGO held the following information: (i) the gene universe, functionally annotated with GO terms, (ii) the gene score “*p* value” for climate association, (iii) the gene-to-GO term(s) mapping, and (iv) the GO hierarchy to account for structure among GO terms, provided by the GO.db package v3.8.2 (Carlson, 2019). The ontology parameter was set to investigate biological processes, molecular functions, or cellular components individually. The node size pruned GO terms with less than 10 annotated genes. The default weight01 algorithm (based on the elim and weight methods, Alexa et al., 2006) and Kolmogorov–Smirnov (KS) test statistic were implemented. For each enrichment test, the *p* values computed by the default “weight01” method were unadjusted for multiple testing. Multiple testing correcting procedures may be invalid because of the nonindependence of the GO term hierarchy (the *p* value of a GO term is conditioned on the neighboring terms) and with the annotation of multiple GO terms to one gene (Skarman et al., 2009).

## Results

### Climate data PCA

The climate data set of 22 variables was reduced in dimensionality to four PCs (Supplementary Figure S1) which captured 92.4% of the variance (38.7%, 33.2%, 14.5%, and 6.0%, respectively). Hence, the reported results will focus primarily on PC1 and PC2, with PC3 and PC4 results available in the supplementary material. The key contributing variables to the four climate variables were identified as follows: temperature across the year (PC1), precipitation in the driest and warmest periods (PC2), precipitation in the wettest months (PC3), and wind speed, precipitation seasonality, and daily temperature range (PC4). Further details are provided in Supplementary Figures S2–S4.

### Whole-genome scan: Detecting loci potentially involved in adaptation to climate

A summary of the covariance matrix, Ω, of SNP allele frequencies is plotted in the supplementary material (Supplementary Figure S5). In general, the population genetic structure is similar to that described in Spurgin et al. (2019), with the Corsica population standing out as the most differentiated from all other populations.

### Identification of outlier loci under selection

The population differentiation analysis using XtX statistics, simulated XtX values of 400,000 neutral SNPs, to infer a 1% XtXPOD_400,000_ significance threshold of 25.88. Across the three runs of the core model, which gave similar results, a total of 4,823 variants were identified as XtX outliers (Supplementary Figure S6). These SNPs were situated in or near 1,535 unique genes, which between them had 2,730 annotations (GO terms). The number of genes and annotations is not expected to be the same because some genes have multiple annotations, and some annotations are associated with multiple genes.

### Genome-environment analysis (GEA): Identification of genes putatively involved with climate adaptation

**Figure 2.**
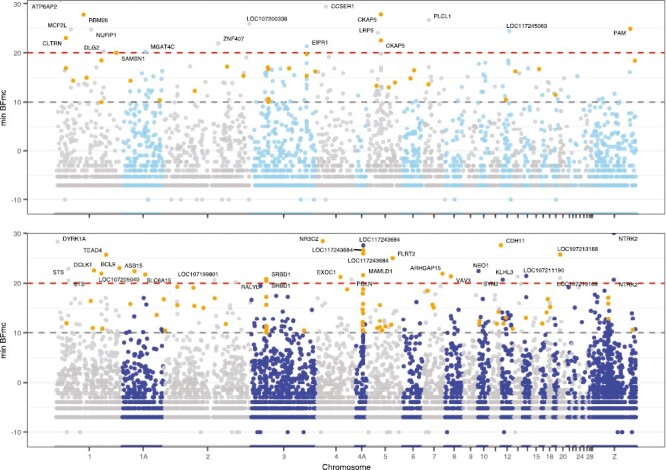
Manhattan plot of annotated genes associated with climate adaptation for (A) PC1 and (B) PC2 at the decisive evidence threshold (BFmc > 20). For all annotated variants, the minimum BFmc score across the three runs is shown. The strong (BFmc > 10), very strong (BFmc > 15), and decisive evidence thresholds are indicated by dashed lines. SNPs highlighted orange (at threshold BFmc > 10) were also found to be under selection in the outlier locus analyses that did not consider climatic data.

At the decisive evidence level (BF ≥ 20 dB), 37 and 67 variants were associated with PC1 and PC2, respectively (Supplementary Table S3). A total of 18 (PC1) and 32 (PC2) SNPs were found in annotated genes, of which 6 and 18 SNPs—more than expected by chance (binomial test, *p* < 2.2 × 10^−16^ for PC1 and PC2, respectively)—were also outliers in the core model (Figure 2). Details of each identified and annotated SNP are reported for all four climate PCs in the supplementary material (Supplementary Table S4, Supplementary Figures S7 and S8). Allele frequencies in each population at the strongest candidate SNPs potentially involved in climate adaptation are included in Supplementary Figure S9 (PC1) and Supplementary Figure S10 (PC2).

### Gene Ontology (GO) term enrichment analysis

#### Biological process GO term enrichment—PC1

A total of 4,428 GO terms found in 7,674 annotated genes were associated with biological processes. A total of 41 biological process GO terms were significantly (*p* < .01) enriched within genes associated with PC1. The most enriched GO term (GO:0061178) is involved in insulin secretion in the cellular response to glucose (Supplementary Table S5, Data file_PC1.csv). There was one candidate gene, CLTRN, that is potentially associated with climate adaptation (see Supplementary Table S7) with an SNP that is significant for both the BF and XtX BayPass statistics and has enriched GO terms (regulation of biological quality GO:0065008 and regulation of transporter activity GO:0032409).

#### Biological process GO term enrichment—PC2

For PC2, 29 biological process GO terms were significantly (*p* < .01) enriched. The two most enriched GO terms were involved in plasma lipid clearance (GO:0034381) and eating behavior (GO:0042755), see Supplementary Table S6, Data File GOtoGene_PC2.txt.

The enriched GO term ATP biosynthetic process (GO:0006754) was notable for being an annotation of genes with the SNPs that were significantly associated with both PC1 and PC2. Details for PCs 3 and 4, 28 and 23 significantly (*p* < .01) enriched biological process GO terms were identified (Supplementary Tables S9 and S10, files GotoGene_PC3.txt and GotoGene_PC4.txt). Biological process GO terms over-represented across any two or more of the PCs are provided in Supplementary Table S8.

#### Molecular function GO term enrichment test

For PC1–PC4, respectively, 12, 8, 14, and 3 out of 740 molecular function GO terms were significantly (*p* < .01) enriched (Supplementary Tables S11–S14).

#### Cellular component GO term enrichment test

For PC1–PC4, respectively, 11, 11, 6, and 6 out of 505 cellular component GO terms were identified as significantly (*p* < .01) enriched (Supplementary Tables S15–S18).

## Discussion

We report numerous genes and pathways in wild populations of great tits that are associated with a wide range of climate variables. The genes are spread across the genome, and there is a segregating genetic variation within populations in these regions. There are no individual regions of the genome that appear to have undergone dramatic changes, such that populations experiencing the most extreme climatic conditions are fixed for putative positively selected alleles or haplotypes. Taken together, these observations suggest that local adaptation to past and present climate conditions is genetically complex, and has occurred, at least partially, through microevolutionary change. Given the polygenic nature of climate adaptation, individual loci have probably had small phenotypic effects. The data indicate that there remains substantial genetic variation associated with climate adaptation within populations. However, despite the weak genome-wide genetic structure across great tit populations, putative climate-adaptation regions do show some between-population differentiation. Given the considerable number of genes and pathways involved, climate adaptation loci are likely to be a large mutational target (Bay et al., 2018). That, and the presence of within- and between-population genetic variation in loci that possibly cause adaptation means there is the potential for great tits to adapt to future climate changes.

This is the first study to specifically look for genes associated with climate adaptation in this species. However, in the same wild great tit populations, Spurgin et al. (2019) reported selection acting on genes thought to be associated with variation in morphology, stress response, and coloration, with adaptation possibly strongest at the range edges of the species’ distribution. The gene *CALM2* (biological function: calcium ion binding) was identified as a locus under selection in Spurgin et al. (2019) and in this study (here, it was associated with PC3 and PC4). Furthermore, *CALM2* has been reported as a candidate gene for desert adaptation in sheep (Yang et al., 2016); in sheep, it is involved in renal epithelial cell processes of water regulation and natriuresis (sodium excretion to stabilize blood pressure). Spurgin et al. (2019) found *CALM2* to be under-selection in the English and Spanish populations. Here it was identified as the strongest candidate gene (i.e., greatest BF) for adaptation to variables influencing PC3 (associated with rain) and PC4 (associated with wind speed, rain seasonality, and daily temperature range) (Supplementary Table S4). Thus, this locus might be under selection in response to climatic conditions. However, none of the other outlier variants in Spurgin et al. (2019) were co-identified in this study, suggesting that signatures of selection at those loci result from adaptation to other selective pressures.

Similarly, a recent study identified loci under divergent selection between Wytham Woods (UK) and two Dutch great tit populations (Bosse et al., 2017). The highly significant outlier regions contained 28 annotated candidate genes involved in skeletal development, morphogenesis, and palate development. These loci were suggested to be involved in the adaptive evolution of longer bills in UK populations, most notably at the COL4A5 gene. In our study, two of the best candidate genes potentially associated with climate adaptation found were also among significant outliers in the Bosse et al. (2017) study; two SNPs were identified in/near *SRBD1* (biological function: mRNA binding) associated with PC2, while eight SNPs were identified in/near *TRPS1* (biological function: cranioskeletal development) associated with PC3. Climate may affect what food sources are available, and that in turn may affect the selection of beak and/or skull shape for foraging performance. For example, in Darwin’s finches, a severe El Nino event led to a scarcity of large seeds, which favored selection on small beak sizes and led to an evolutionary change (Grant & Grant, 1993). Thus, climate adaptation may be the explanation for signatures of selection at these two loci, but more generally, there is not much overlap between the potential climate adaptation loci reported here, and previous attempts to find genes under selection in these populations. Of course, that may be because this study explores populations experiencing a much wider range of climatic variation. Incorporating other possible agents of selection into GEA models could enhance our understanding of what is shaping genomic variation at other highly differentiated loci.

A key question is whether the same suite of genes are involved in adaptation to climate in different species. We directly compared the set of genes found to be “decisively” associated with climate in great tit, with 12 positively selected heat-stress-adaptation genes identified from a similar analysis in chicken populations experiencing desert and monsoon island climates (Tian et al., 2020). In our study, nine of the 12 genes had an SNP within 2.5kbp, but none were associated with climate adaptation. However, two of the genes (*ADCY1* and *VPS13C*) were identified in the pathways of enriched biological process GO terms (for PC1–4 and PC4, respectively) associated with climate adaptation in great tits (Supplementary Table S19). Adaptation to heat stress has previously been demonstrated in genes linked to the arachidonic acid metabolism pathway in chickens (Tian et al., 2020), with CYP2J21 and CYP2J23 upregulated in pectoral muscles of heat-stress-treated broiler chickens, with oxidative stress recorded in the pectoral muscle (Liu et al., 2022). Two of those arachidonic acid metabolism genes (*DRD3* and *TNFRSF11A*) are annotated with one of the GO terms (GO:0065008 “regulation of biological quality”) that were was associated with PC1 in this study (Supplementary Table S19). Caizergues et al. (2022) identified a significant change in methylation linked to the DRD3 gene in urban populations of great tit compared to forest environments. Extreme heating events in urban areas may lead to heat stress for nestlings and adults (Sumasgutner et al., 2023). Heat stress adaptation has previously been linked with convergent evolution of four genes (*PLA2G12b*, *GRP17, TNFRSF11A*, and *OC90*) between mammals and chickens (Tian et al., 2020). Of these genes, only *OC90* was co-identified in this GEA study, where it was associated with climate (PC4). There were no SNPs within 2.5kbp of *GRP17.* In our analyses, PC2 is the PC most influenced by climate variables that are associated with extreme heat (Supplementary Figure S2). There is no overlap between genes associated with PC2 and the heat-stress-associated genes in the studies described earlier.

There is little overlap between the “decisive” candidate climate adaptation genes found here to those found in similar studies of more distantly related (i.e., non-avian) taxa such as *Drosophila* (Bogaerts-Márquez et al., 2021) and cattle (Flori et al., 2019). An exception is the gene *BTBD7*, which is involved in protein ubiquitination, and was identified as a climate adaptation locus in *Drosophila* (Bogaerts-Márquez et al., 2021). Here, *BTBD7* is associated with PC3 (Supplementary Table S20). Avian cells respond to temperature-induced heat stress, via stimulated protein ubiquitination (Pritchett et al., 2023). Similarly, there is some evidence of overlap of the climate-associated enriched GO terms identified here with those in cattle (Flori et al., 2019); these GO terms are associated with cellular signaling and nervous system function.

For both PC1 and PC2, the most strongly over-represented GO terms were not terms where we could find evidence of climate adaptation in other species. For PC1, the top-ranked GO term was “regulation of insulin secretion.” This term may potentially be linked to climate-related effects on diet and hormones. For example, cold temperatures are thought to prime the glucose stress response in tree swallows (Ryan et al., 2023). For climate PC2, the top-ranked GO term is “plasma lipoprotein particle clearance” (involved in the release of cholesterol). Climate variation may influence changes in food availability and nestling growth (Keller & Noordwijk, 1994). Furthermore, poor nutrition in great tit embryos and early life stage may constrain development and adult health and fitness (Toledo et al., 2016). Thus, it is possible that variations in genes involved in pathways that affect plasma lipoproteins have provided an adaptive response to harmful climatic effects on nutrition.

Overall, the patterns suggest that climate adaptation gene discovery studies have identified different candidate genes that might be involved in adaptation, but that these candidates have some shared gene pathways for biological processes. There may be so little overlap because of the very different investigated taxa (invertebrates, domesticated livestock, and birds), and also because of the very different physiological routes to climate adaptation (e.g., morphology, behavior, physiology, neuro-endocrine, biochemical, metabolism, cellular, and molecular responses). In addition, different genes with related functions may enable adaptation to similar environments. Finally, when conducting GEA studies, it is necessary to be mindful that some “hits” might be false positives, which are not expected to overlap between different independent studies. One take-home message from this article is that identifying individual loci that are definitively associated with climate adaptation is challenging. The combination of multiple climate-related variables driving selection, a polygenic genetic architecture of the (unknown) traits under selection, a lack of good candidates from other systems, and an absence of selected loci going to fixation in specific populations all contribute to making gene discovery difficult.

Best practice for the confirmation of candidate genes from genome-environment analysis should ideally include an experimental verification, for example, by functional genomics or gene expression studies (Rellstab et al., 2015). While beyond the scope of this study, such an approach is feasible in wild great tit populations, for example, by rearing and selective breeding of wild-caught individuals in aviaries where environmental conditions can be controlled. For example, Gienapp et al. (2019) selected for differences in lay date in aviary birds, and then looked at the fitness of early and late layers when they were moved as eggs to nest boxes in the forest.

Methods to reliably forecast how populations may adapt to climate change (changes in allele frequencies) and how climate adaptation genes may be maintained across populations (population differentiation), will require testing of how observable (and possibly repeatable) the genetic effects described here are. Ongoing monitoring of allele frequencies in the study populations, in tandem with an increase in the number of individuals genotyped per population would improve power, as would the inclusion of data from additional populations. For those populations where climate change has been documented and samples are available over many years, it may be possible to look for associations between genotypes at climate-related SNPs and variation in fitness traits. In particular, it could be asked whether any selection at these SNPs varies temporally, in a way predicted by within-population changes in climatic conditions (PC1–4). The identity and location of sites under selection could be improved with whole genome sequencing (which is now becoming much closer in cost to SNP chip genotyping). To validate the candidate genes that have possibly enabled climate adaptation, and to date the timing of adaptive changes, it should be possible to investigate the genomes of museum samples, and formally test whether signatures of selection can be identified in the same genes over the last few hundred years of climate change (Clark et al., 2023).

Finally, it is possible that identifying adaptive genomic variation to climate variation may aid our understanding of the climatic drivers of population declines and genomic vulnerabilities. In summary, we found evidence that great tits have adapted over historical times to different climates through numerous evolutionary changes and that the process is complicated, caused by the selection at many different genes, probably driven by many different climatic variables. Most of the genes reported here have not previously been reported to be associated with climate adaptation. Given that many of the SNPs tagging regions responsible for adaptation are still segregating within populations, it seems likely that there remains substantial genetic variation within and between populations that can help further adaptation to future climate changes.

## Supplementary Material

qrad043_suppl_Supplementary_Tables_S1_S20_Figures_S1_S10

qrad043_suppl_Supplementary_GOtoGene_PC1

qrad043_suppl_Supplementary_GOtoGene_PC2

qrad043_suppl_Supplementary_GOtoGene_PC3

qrad043_suppl_Supplementary_GOtoGene_PC4

## Data Availability

Data and scripts are available at: https://doi.org/10.5061/dryad.vt4b8gtxd (Stonehouse et al. 2023).
