# Supplementary material for: The genomics of adaptation to climate in European great tit (Parus major) populations
Source: Evol Lett. 2023 Oct 12;8(1):18–28. doi: 10.1093/evlett/qrad043 (PMC10872194; doi:10.1093/evlett/qrad043)
Supplement: qrad043_suppl_Supplementary_Tables_S1_S20_Figures_S1_S10 [file qrad043_suppl_supplementary_tables_s1_s20_figures_s1_s10.pdf]

Supplementary material

Table S1: Sample sizes and locations (decimal grid reference to 2 d.p.) of the 20 study populations.

Popn ID	Population	Latitude	Longitude	Individuals
1	Loch Lomond (Scotland)	56.13	-4.62	19
2	Cambridge (UK)	52.40	-0.23	27
3	Wytham (UK)	51.77	-1.34	47
4	Mariola (Spain)	38.73	-0.55	19
5	Montpellier (France)	43.61	3.87	32
6	Antwerp (Belgium)	51.13	4.53	15
7	Westerheide (NL)	52.00	5.83	48
8	Vlieland (NL)	53.28	5.01	15
9	Zurich (Switzerland)	47.39	8.57	21
10	Radolfzell (Germany)	47.74	8.98	17
11	Seewisen (Germany)	47.96	11.18	30
12	Muro (Corsica)	42.55	8.92	20
13	Vienna (Austria)	48.21	16.26	27
14	Velky Kosir (Czech Republic)	49.54	17.07	21
15	Pilis Mountains (Hungary)	47.72	19.02	28
16	Gotland (Sweden)	57.14	18.33	30
17	Harjavalta (Finland)	61.33	22.17	43
18	Oulu (Finland)	65.13	25.88	29
19	Tartu (Estonia)	58.12	25.08	30
20	Zvenigorod (Russia)	55.73	36.85	17

Table S2: Bioclimate variables extracted from WorldClimv2 at 30sec spatial resolution, (approx. $1km^2$ surface area). This data set has average monthly climate data for min, mean and max temp and precipitation, and uses data from 1970-2000.

Identifier	Bioclimate_Variable	Unit
BIO1	Annual Mean Temperature	°C
BIO2	Mean Diurnal Range (Mean of monthly (max temp - min temp)	°C
BIO3	Isothermality (BIO2/BIO7) (* 100) - Diurnal Range / Annual Range * 100	°C
BIO4	Temperature Seasonality (standard deviation *100)	sd
BIO5	Max Temperature of Warmest Month	°C
BIO6	Min Temperature of Coldest Month	°C
BIO7	Temperature Annual Range (BIO5-BIO6)	°C
BIO8	Mean Temperature of Wettest Quarter	°C
BIO9	Mean Temperature of Driest Quarter	°C
BIO10	Mean Temperature of Warmest Quarter	°C
BIO11	Mean Temperature of Coldest Quarter	°C
BIO12	Annual Precipitation	mm
BIO13	Precipitation of Wettest Month	mm
BIO14	Precipitation of Driest Month	mm
BIO15	Precipitation Seasonality	Coefficient of variation
BIO16	Precipitation of Wettest Quarter	mm
BIO17	Precipitation of Driest Quarter	mm
BIO18	Precipitation of Warmest Quarter	mm
BIO19	Precipitation of Coldest Quarter	mm
srad	solar radiation	kJ m-2 day-1
wind	wind speed	m s-1
vapr	water vapor pressure	kPa

Table S3: The number of variants associated with climate adaptation at the decisive, and strong levels and above, for raw and annotated data for PCs 1-4. The number of 'decisive' climate-associated variants also directly identified as 'outlier' variants are indicated in brackets.

	Decisive	Strong
PC1raw	37 (11)	292
PC1ann	18 (6)	179 (36)
PC2raw	67 (32)	506
PC2ann	32 (18)	275 (76)
PC3raw	45 (21)	329
PC3ann	32 (20)	204 (49)
PC4raw	101 (13)	711
PC4ann	54 (5)	407 (42)

Table S4: Annotated variants associated with climate adaptation for PC1-PC4, listed in order of genome location (chromosome and position in base pairs). Information on the identified gene ID and gene symbol are displayed, with the averaged BFmc score over the 3 runs. Variants co-identified as outliers under selection by XtX score or in a gene with a variant identified under selection are indicated by * or / respectively.

Chr	Pos	PC	Gene.Id	Gene.Sym	A1/A2	Av.BFmc	XtX.Outlier
1	2,565,575	PC2	107204791	DYRK1A	C/T	30.42	
1	7,518,315	PC1	107212355	ATP6AP2	A/G	38.35	
1	10,270,551	PC4	107201519	DMD	C/T	51.96	*
1	13,884,888	PC4	107201818	PHEX	A/C	22.59	
1	16,831,257	PC1	107202978	CLTRN	A/G	24.57	*
1	21,091,359	PC2	107204502	STS	A/G	27.97	/
1	21,091,407	PC2	107204502	STS	A/G	23.76	/
1	25,405,731	PC1	107207436	MCF2L	C/T	29.36	/
1	25,824,967	PC3	107208751	TMEM255B	T/C	32.44	*
1	41,024,831	PC4	107211211	GPC6	C/T	45.00	/
1	41,024,917	PC4	107211211	GPC6	G/A	34.62	/
1	47,206,649	PC1	107203556	RBM26	C/A	29.18	*
1	58,853,250	PC4	107206861	DGKH	A/G	26.14	/
1	60,174,414	PC1	107207483	NUFIP1	G/A	28.54	
1	63,530,963	PC4	107208330	LHFPL6	C/T	41.65	/
1	65,154,295	PC2	107206363	DCLK1	C/G	24.93	*
1	71,327,611	PC3	107203451	ZMYM2	G/A	29.72	/
1	74,778,324	PC4	107209500	YAP1	A/G	34.03	
1	77,711,671	PC2	107205040	LOC107205040	A/T	23.14	*
1	79,834,444	PC4	107207537	GRM5	G/C	45.44	
1	81,418,748	PC1	107200839	DLG2	T/C	21.12	/
1	86,031,767	PC2	107211077	TEAD4	G/C	30.99	*
1	103,524,064	PC1	107210090	SAMSN1	G/T	22.84	*
1	108,854,195	PC2	107208972	BCL9	C/A	24.79	*
2	64,321,336	PC4	107200567	LOC107200567	T/C	25.07	/
2	87,332,777	PC2	107199801	LOC107199801	C/G	21.55	/
2	93,611,282	PC1	107200124	ZNF407	T/C	23.91	/
2	124,047,383	PC2	107200779	RALYL	C/A	21.11	/
2	129,906,821	PC3	107199830	VPS13B	A/G	44.66	*
2	133,581,783	PC3	117243976	LOC117243976	C/T	30.28	*
2	133,583,850	PC3	117243976	LOC117243976	G/A	49.62	*
2	133,774,454	PC4	107200135	SYBU	C/G	29.55	
2	135,146,669	PC4	107200494	CSMD3	A/G	41.57	/
2	136,082,831	PC3	107200727	TRPS1	A/G	48.95	*
2	136,083,734	PC3	107200727	TRPS1	T/C	46.11	*
2	136,084,949	PC3	107200727	TRPS1	C/T	48.02	*
2	136,091,965	PC3	107200727	TRPS1	A/T	23.78	*
2	136,134,403	PC3	107200727	TRPS1	A/G	27.67	*
2	136,162,421	PC3	107200727	TRPS1	C/T	21.96	*
2	136,162,978	PC3	107200727	TRPS1	C/T	30.08	*

Table S4: Annotated variants associated with climate adaptation for PC1-PC4. (*Continued*).

Chr	Pos	PC	Gene.Id	Gene.Sym	A1/A2	Av.BFmc	XtX.Outlier
2	136,185,927	PC3	107200727	TRPS1	A/G	23.50	*
2	138,279,950	PC4	107199730	SNTB1	T/C	23.62	
2	142,623,087	PC4	107198490	OC90	A/C	28.91	
2	147,182,881	PC1	107200338	LOC107200338	T/C	27.11	/
3	23,360,975	PC4	107201852	EML4	T/C	24.50	/
3	24,847,522	PC4	107201772	THADA	G/C	20.55	
3	25,925,802	PC2	107202354	SRBD1	G/A	24.02	*
3	26,021,155	PC2	107202354	SRBD1	T/C	22.45	*
3	28,640,451	PC3	107201314	CALM2	T/G	24.29	*
3	28,640,451	PC4	107201314	CALM2	T/G	30.05	*
3	48,808,733	PC3	107201815	UTRN	A/T	34.94	
3	48,808,845	PC3	107201815	UTRN	A/G	29.19	
3	48,808,975	PC3	107201815	UTRN	A/G	28.51	
3	64,276,305	PC4	107202074	MCM9	G/A	24.74	/
3	65,270,599	PC4	117244053	LOC117244053	A/G	21.94	
3	95,364,958	PC1	107202206	EIPR1	A/G	23.12	/
3	104,023,997	PC4	107202237	LDAH	G/A	22.00	
3	111,336,394	PC4	107201996	EXTL3	T/C	51.96	
4	8,364,199	PC4	107203104	SCOC	G/A	26.34	
4	8,976,253	PC3	107203153	INPP4B	T/C	23.06	/
4	11,377,682	PC2	107202999	NR3C2	T/C	30.74	*
4	16,861,971	PC1	107203391	CCSER1	C/T	32.45	/
4	21,077,788	PC4	117244290	LOC117244290	C/G	24.24	
4	40,511,106	PC4	107203403	TUSC3	G/A	32.71	/
4	41,856,879	PC2	107203327	EXOC1	T/C	27.91	*
4	50,984,772	PC3	107203565	LGI2	T/C	23.39	
4	60,662,794	PC2	107203258	POLN	A/G	21.65	
4	62,155,864	PC4	107203239	UVSSA	A/C	28.29	/
5	14,615,044	PC4	107206273	KCNQ1	G/A	25.29	/
5	17,982,769	PC1	107206074	LRP5	C/G	29.34	
5	18,049,957	PC4	107205715	PPP6R3	C/T	23.11	
5	23,310,278	PC1	107205919	CKAP5	A/G	25.30	*
5	23,311,729	PC1	107205919	CKAP5	C/T	35.58	*
5	25,223,763	PC4	107206045	MAPKBP1	G/A	22.10	
5	37,242,989	PC4	107206166	BRMS1L	A/G	40.74	/
5	43,487,765	PC2	107205788	FLRT2	G/A	29.60	*
5	46,653,295	PC3	107205492	BTBD7	T/C	43.44	*
6	2,869,379	PC4	107207071	PRKG1	T/C	30.08	
6	13,025,966	PC4	107206768	LOC107206768	A/G	27.10	*
6	15,294,049	PC4	107207110	LOC107207110	G/A	24.26	
7	6,718,967	PC4	107207630	GULP1	A/C	44.49	*
7	6,725,737	PC3	107207630	GULP1	C/T	23.31	*
7	10,009,285	PC1	107207496	PLCL1	C/T	28.95	/
7	11,380,293	PC4	107207396	BMPR2	G/A	46.08	/
7	13,532,269	PC4	107207465	DNAJC10	C/A	30.20	

Table S4: Annotated variants associated with climate adaptation for PC1-PC4. (*Continued*).

Chr	Pos	PC	Gene.Id	Gene.Sym	A1/A2	Av.BFmc	XtX.Outlier
7	21,380,676	PC4	107207460	RBMS1	T/C	22.61	
7	27,580,666	PC4	107207555	MYLK	C/T	25.85	/
7	33,081,327	PC4	107207704	LRP1B	G/A	28.41	
7	33,564,968	PC2	107207713	ARHGAP15	A/G	22.67	*
8	3,171,789	PC4	107208299	RASAL2	A/G	29.41	*
8	5,677,334	PC4	107208208	PLA2G4A	G/A	28.78	/
8	10,360,170	PC2	107208342	VAV3	T/C	25.67	*
9	3,692,204	PC4	107208868	COPB2	T/C	37.23	/
10	1,348,966	PC2	107209331	NEO1	T/G	24.17	/
10	9,569,304	PC3	107209391	DUT	G/T	30.78	
11	18,643,057	PC3	107209916	UBA2	C/T	27.48	*
11	19,793,190	PC2	107209891	CDH11	T/C	29.59	*
12	2,805,249	PC2	107210106	SYN2	C/T	22.00	/
12	14,106,471	PC1	117245063	LOC117245063	T/C	30.06	
12	16,782,382	PC4	107210088	PROK2	G/A	33.79	
12	17,455,863	PC4	107210404	LOC107210404	T/C	36.34	
13	7,172,536	PC4	107210556	ADAMTS2	C/G	26.73	/
13	7,983,864	PC2	107210622	KLHL3	C/G	22.84	/
14	6,280,798	PC2	107211190	LOC107211190	T/G	31.30	
15	8,308,275	PC4	107211593	LOC107211593	G/A	25.54	
15	10,103,945	PC3	107211648	CIT	C/T	36.08	
15	10,634,483	PC3	107211537	RNF185	A/G	27.12	*
18	3,238,687	PC4	107212330	HELZ	A/G	27.13	
18	4,435,759	PC4	107212395	ANKFN1	G/A	20.87	
19	7,609,632	PC4	107212793	BCAS3	A/G	27.44	/
20	2,578,923	PC2	107213168	LOC107213168	T/C	22.67	/
20	2,894,619	PC2	107213168	LOC107213168	T/C	33.01	*
23	757,962	PC4	107214008	OPRD1	T/C	25.75	
28	3,677,144	PC4	107215475	ARMC6	T/A	44.78	
1A	20,577,550	PC2	109022637	ASB15	C/G	23.20	*
1A	35,501,527	PC4	107204504	KCNC2	G/A	24.18	
1A	36,689,589	PC3	107204773	NAV3	A/G	27.26	*
1A	39,314,068	PC2	107204954	SLC6A15	A/G	24.25	*
1A	39,895,014	PC1	107205204	MGAT4C	C/T	23.49	
4A	12,856,478	PC2	107203727	MAMLD1	T/C	23.37	*
4A	12,895,120	PC2	107203727	LOC117243684	C/T	31.02	*
4A	12,913,433	PC2	107203727	LOC117243684	A/C	26.91	*
4A	13,024,266	PC2	107203727	LOC117243684	T/A	29.66	/
Z	6,392,305	PC3	107216348	CELF4	A/C	25.92	
Z	7,925,628	PC4	107198493	CNTFR	C/T	25.69	
Z	15,964,452	PC4	107216157	ARL15	T/C	25.70	
Z	34,137,890	PC3	107216294	GNAQ	T/C	42.69	
Z	34,804,830	PC3	107198365	TLE4	G/A	29.31	
Z	36,454,158	PC2	107216220	NTRK2	T/C	33.18	
Z	36,455,313	PC2	107216220	NTRK2	T/A	24.04	

Table S4: Annotated variants associated with climate adaptation for PC1-PC4. (*Continued*).

Chr	Pos	PC	Gene.Id	Gene.Sym	A1/A2	Av.BFmc	XtX.Outlier
Z	43,967,033	PC3	107216081	LOC107216081	T/G	32.08	*
Z	50,623,277	PC4	107198410	MUSK	C/G	29.82	
Z	64,810,286	PC1	107215969	PAM	A/T	25.39	*
Z	69,336,303	PC3	107198258	CAST	C/G	37.37	*
Z	73,364,875	PC3	107216310	ATG10	G/T	42.92	
Z	73,486,535	PC4	107216169	SSBP2	T/C	23.52	

Table S5: Biological process GO terms that were significantly enriched among loci associated with PC1. The highest level category of each term is listed. For each term the number of annotated genes considered in the dataset (Ann), the number of annotated genes significantly associated with PC1 (Sign) and the number of expected genes to be associated with PC1 (Exp) are indicated. Statistical significance was determined using a Kolmogorov-Smirnov (KS) test statistic and topGO's default algorithm.

GO.ID	Term	Ann	Sign	Exp	P-val
GO:0061178	regulation of insulin secretion involved in cellular response to glucose stimulus	28	1	0.22	0.00004
GO:0062014)	negative regulation of small molecule metabolic process	42	0	0.33	0.00025
GO:0001829	trophectodermal cell differentiation	12	0	0.09	0.00034
GO:0007507	heart development	283	5	2.21	0.00040
GO:0140013	meiotic nuclear division	67	1	0.52	0.00079
GO:0007368	determination of left/right symmetry	59	1	0.46	0.00103
GO:0032924	activin receptor signaling pathway	23	0	0.18	0.00159
GO:0097178	ruffle assembly	21	0	0.16	0.00229
GO:0065008	regulation of biological quality	1602	13	12.53	0.00240
GO:0032467	positive regulation of cytokinesis	19	0	0.15	0.00244
GO:0006403	RNA localization	60	0	0.47	0.00296
GO:0046685	response to arsenic-containing substance	10	1	0.08	0.00314
GO:0031297	replication fork processing	25	0	0.20	0.00317
GO:0045332	phospholipid translocation	18	0	0.14	0.00353
GO:0009063	cellular amino acid catabolic process	35	0	0.27	0.00385
GO:0044827	modulation by host of viral genome replication	12	0	0.09	0.00390
GO:0034660	ncRNA metabolic process	233	2	1.82	0.00429
GO:0008630	intrinsic apoptotic signaling pathway in response to DNA damage	42	0	0.33	0.00471
GO:0048563	post-embryonic animal organ morphogenesis	10	0	0.08	0.00483
GO:003132	positive regulation of cellular metabolism	1449	11	11.33	0.00538

Table S5: Significantly enriched GO terms for biological process – PC1 (*Continued*).

GO.ID	Term	Ann	Sign	Exp	P-val
GO:0048384	retinoic acid receptor signaling pathway	12	0	0.09	0.00565
GO:0010875	positive regulation of cholesterol efflux	10	0	0.08	0.00566
GO:0097154	GABAergic neuron differentiation	11	0	0.09	0.00566
GO:0048534	hematopoietic or lymphoid organ development	428	4	3.35	0.00573
GO:0021954	central nervous system neuron development	48	0	0.38	0.00575
GO:0002027	regulation of heart rate	47	1	0.37	0.00598
GO:0060324	face development	34	1	0.27	0.00666
GO:0051897	positive regulation of protein kinase B signaling	46	2	0.36	0.00733
GO:0006754	ATP biosynthetic process	16	1	0.13	0.00764
GO:0009067	aspartate family amino acid biosynthetic process	10	0	0.08	0.00776
GO:0032409	regulation of transporter activity	111	1	0.87	0.00833
GO:0006119	oxidative phosphorylation	34	1	0.27	0.00834
GO:0007178	transmembrane receptor protein serine/threonine kinase signaling pathway	190	1	1.49	0.00855
GO:0018958	phenol-containing compound metabolic process	48	2	0.38	0.00865
GO:0033260	nuclear DNA replication	13	0	0.10	0.00884
GO:0006413	translational initiation	55	0	0.43	0.00919
GO:0046033	AMP metabolic process	14	0	0.11	0.00921
GO:0045910	negative regulation of DNA recombination	23	0	0.18	0.00927
GO:0099601	regulation of neurotransmitter receptor activity	19	0	0.15	0.00937
GO:0010469	regulation of signaling receptor activity	55	0	0.43	0.00964
GO:0006893	Golgi to plasma membrane transport	33	0	0.26	0.00965

Table S6: Biological process GO terms that were significantly enriched among loci associated with PC2. Statistical significance was determined using a Kolmogorov-Smirnov (KS) test statistic and topGO's default algorithm (details as for Table S5).

GO.ID	Term	Ann	Sign	Exp	P-val
GO:0034381	plasma lipoprotein particle clearance	12	0	0.09	0.00043
GO:0007613	memory	47	0	0.35	0.00085
GO:0006814	sodium ion transport	74	1	0.55	0.00097
GO:0042755	eating behavior	11	0	0.08	0.00121
GO:0042177	negative regulation of protein catabolic process	61	1	0.45	0.00152
GO:0034656	nucleobase-containing small molecule catabolic process	13	2	0.10	0.00365
GO:0070129	regulation of mitochondrial translation	11	0	0.08	0.00368
GO:0048167	regulation of synaptic plasticity	66	0	0.49	0.00396
GO:0032647	regulation of interferon-alpha production	13	1	0.10	0.00405
GO:0006833	water transport	10	0	0.07	0.00411
GO:0051603	proteolysis involved in cellular protein catabolic process	349	3	2.59	0.00443
GO:0021544	subpallium development	19	0	0.14	0.00448
GO:0051726	regulation of cell cycle	477	5	3.54	0.00502
GO:0044706	multi-multicellular organism process	55	3	0.41	0.00519
GO:0008038	neuron recognition	13	1	0.10	0.00547
GO:0006754	ATP biosynthetic process	16	0	0.12	0.00615
GO:0021515	cell differentiation in spinal cord	25	0	0.19	0.00633
GO:0043931	ossification involved in bone maturation	10	0	0.07	0.00728
GO:0022617	extracellular matrix disassembly	11	0	0.08	0.00739
GO:0071637	regulation of monocyte chemotactic protein-1 production	11	0	0.08	0.00785
GO:0008203	cholesterol metabolic process	35	0	0.26	0.00805
GO:0006956	complement activation	15	0	0.11	0.00807
GO:0010633	negative regulation of epithelial cell migration	34	0	0.25	0.00817
GO:0016197	endosomal transport	101	1	0.75	0.00855

Table S6: Significantly enriched GO terms for biological process – PC2 (*Continued*).

GO.ID	Term	Ann	Sign	Exp	P-val
GO:0060088	auditory receptor cell stereocilium organization	12	2	0.09	0.00868
GO:0070646	protein modification by small protein removal	91	0	0.68	0.00870
GO:0051253	negative regulation of RNA metabolic process	599	3	4.45	0.00924
GO:0050714	positive regulation of protein secretion	65	2	0.48	0.00994
GO:0140694	non-membrane-bounded organelle assembly	176	1	1.31	0.00997

Table S7: Candidate climate adaptation genes that were identified in significantly enriched GO term pathways associated with each of PC1-4 are presented. The "XtX Outlier" column also indicates if variants were also significant in the core model (*), or if other variants in the same gene were also significant in the core model (/).

PC	Gene	XtX outlier	GO term(s)
PC1	ATP6AP2		regulation of biological quality (GO:0065008)
PC1	CLTRN	*	regulation of biological quality (GO:0065008), regulation of transporter activity (GO:0032409)
PC1	LRP5		regulation of insulin secretion (GO:0061178), regulation of biological quality (GO:0065008), positive regulation of cellular metabolic process (GO:0031325), hematopoietic or lymphoid organ development (GO:0048534)
PC1	PLCL1	/	regulation of biological quality (GO:0065008)
PC2	DYRK1A		negative regulation of RNA metabolic process (GO:0051253)
PC2	TEAD4	*	multi-multicellular organism process (GO:0044706)
PC2	EXOC1	*	positive regulation of protein secretion (GO:0050714)
PC2	KLHL3	/	sodium ion transport (GO:0006814), proteolysis involved in protein catabolic process (GO:0051603)
PC2	NTRK2		regulation of synaptic plasticity (GO:0048167)
PC3	ZMYM2	/	regulation of DNA-templated transcription (GO:0006355)
PC3	TRPS1	*	regulation of DNA-templated transcription (GO:0006355)
PC3	CALM2	*	release of sequestered calcium ion into cytosol by endoplasmic reticulum (GO:1903514)
PC3	DUT		pyrimidine nucleoside triphosphate metabolic process (GO:0009147)
PC3	RNF185	*	protein ubiquitination (GO:0016567)
PC3	CELF4		ribonucleoprotein complex assembly (GO:0022618)
PC3	TLE4		regulation of DNA-templated transcription (GO:0006355)
PC4	DMD	*	chemical homeostasis (GO:0048878)
PC4	DGKH	/	G protein-coupled receptor signaling pathway (GO:0007186)
PC4	YAP1		negative regulation of cilium assembly (GO:1902018)
PC4	GRM5		regulation of synaptic plasticity (GO:0048167), G protein-coupled receptor signaling pathway (GO:0007186)
PC4	THADA		chemical homeostasis (GO:0048878)
PC4	CALM2	*	chemical homeostasis (GO:0048878), cell cycle phase transition (GO:0044770)
PC4	EXTL3		proteoglycan biosynthetic process (GO:0030166)
PC4	KCNQ1	/	chemical homeostasis (GO:0048878), adrenergic receptor signaling pathway (GO:0071875), G protein-coupled receptor signaling pathway (GO:0007186)
PC4	BMPR2	/	negative regulation of muscle cell differentiation (GO:0051148), retina development in camera-type eye (GO:0060041)
PC4	OPRD1		G protein-coupled receptor signaling pathway (GO:0007186)

Table S8: Biological process GO terms that were significantly over-represented among candidate adaptation loci for multiple climate PCs.

GO Term ID	GO Term description	PC
GO:0006754	ATP biosynthetic process	1,2
GO:0032924	activin receptor signaling pathway	1,3
GO:0006119	oxidative phosphorylation	1,3
GO:0006413	translational initiation	1,3
GO:0018958	phenol-containing compound metabolic process	1,3
GO:0021544	subpallium development	2,3
GO:0021515	cell differentiation in spinal cord	2,3
GO:0048167	regulation of synaptic plasticity	2,4

Table S9: Biological process GO terms that were significantly enriched among loci associated with PC3. Statistical significance was determined using a Kolmogorov-Smirnov (KS) test statistic and topGO's default algorithm (details as for Table S5).

GO.ID	Term	Annot	Sign	Exp	P-val
GO:0006119	oxidative phosphorylation	34	1	0.23	0.00063
GO:0009147	pyrimidine nucleoside triphosphate metabolic process	13	1	0.09	0.00078
GO:0006413	translational initiation	55	1	0.38	0.00104
GO:0048643	positive regulation of skeletal muscle tissue development	11	1	0.08	0.00114
GO:0035036	sperm-egg recognition	15	1	0.10	0.00117
GO:0018958	phenol-containing compound metabolic process	48	1	0.33	0.00170
GO:0070306	lens fiber cell differentiation	15	0	0.10	0.00270
GO:0008543	fibroblast growth factor receptor signaling pathway	47	0	0.32	0.00359
GO:0019229	regulation of vasoconstriction	21	0	0.15	0.00360
GO:0006486	protein glycosylation	119	3	0.82	0.00436
GO:0006355	regulation of transcription, DNA-templated transcription	1315	2	9.08	0.00473
GO:0002931	response to ischemia	13	0	0.09	0.00482
GO:0016567	protein ubiquitination	314	1	2.17	0.00560
GO:0032924	activin receptor signaling pathway	23	0	0.16	0.00593
GO:0021544	subpallium development	19	0	0.13	0.00653
GO:0034067	protein localization to Golgi apparatus	14	1	0.10	0.00670
GO:0050731	positive regulation of peptidyl-tyrosine phosphorylation	85	1	0.59	0.00699
GO:0006029	proteoglycan metabolic process	58	0	0.40	0.00705
GO:0043433	negative regulation of DNA-binding transcription factor activity	78	1	0.54	0.00732
GO:0008544	epidermis development	140	2	0.97	0.00754
GO:0021987	cerebral cortex development	64	0	0.44	0.00758
GO:0021515	cell differentiation in spinal cord	25	1	0.17	0.00771
GO:1903514	release of sequestered calcium ion into cytosol by endoplasmic reticulum	11	1	0.08	0.00783

Table S9: Biological process GO terms that were significantly enriched among loci associated with PC3. Statistical significance was determined using a Kolmogorov-Smirnov (KS) test statistic and topGO's default algorithm (details as for Table S5). *(continued)*

GO.ID	Term	Annot	Sign	Exp	P-val
GO:0022618	ribonucleoprotein complex assembly	67	0	0.46	0.00789
GO:0010720	positive regulation of cell development	130	0	0.90	0.00818
GO:0033151	V(D)J recombination	11	0	0.08	0.00827
GO:0007568	aging	19	0	0.13	0.00891
GO:0045859	regulation of protein kinase activity	269	0	1.86	0.00925

Table S10: Biological process GO terms that were significantly enriched among loci associated with PC4. Statistical significance was determined using a Kolmogorov-Smirnov (KS) test statistic and topGO's default algorithm (details as for Table S5).

GO.ID	Term	Annot	Sign	Exp	P-val
GO:0009156	ribonucleoside monophosphate biosynthetic process	19	0	0.16	0.00031
GO:1902018	negative regulation of cilium assembly	10	1	0.09	0.00134
GO:0009798	axis specification	43	1	0.37	0.00161
GO:0010506	regulation of autophagy	143	0	1.23	0.00185
GO:0030166	proteoglycan biosynthetic process	40	0	0.34	0.00244
GO:0048878	chemical homeostasis	455	4	3.91	0.00250
GO:0032651	regulation of interleukin-1 beta production	23	1	0.20	0.00377
GO:0000375	RNA splicing, via transesterification reactions	134	1	1.15	0.00424
GO:0046395	carboxylic acid catabolic process	83	2	0.71	0.00427
GO:0051148	negative regulation of muscle cell differentiation	32	1	0.28	0.00441
GO:0033006	regulation of mast cell activation involved in immune response	10	0	0.09	0.00535
GO:0090181	regulation of cholesterol metabolic process	12	0	0.10	0.00558
GO:0046825	regulation of protein export from nucleus	15	0	0.13	0.00633
GO:0009220	pyrimidine ribonucleotide biosynthetic process	11	0	0.09	0.00640
GO:0048167	regulation of synaptic plasticity	66	0	0.57	0.00684
GO:0044770	cell cycle phase transition	246	4	2.12	0.00740
GO:0071875	adrenergic receptor signaling pathway	11	0	0.09	0.00741
GO:0043300	regulation of leukocyte degranulation	11	0	0.09	0.00806
GO:0060041	retina development in camera-type eye	79	1	0.68	0.00814
GO:0007186	G protein-coupled receptor signaling pathway	329	2	2.83	0.00825
GO:1903307	positive regulation of regulated secretory pathway	12	0	0.10	0.00828
GO:0072525	pyridine-containing compound biosynthetic process	12	0	0.10	0.00844
GO:0006111	regulation of gluconeogenesis	21	0	0.18	0.00920

Table S11: Molecular function GO term enrichment (weight01 algorithm and KS statistic) for PC1.

GO.ID	GO Term	Annotated	Significant	Expected	P-val
GO:0140326	ATPase-coupled intramembrane lipid transporter activity	18	0	0.15	0.000098
GO:0005212	structural constituent of eye lens	10	0	0.08	0.00033
GO:0140662	ATP-dependent protein folding chaperone	19	0	0.16	0.00040
GO:0051082	unfolded protein binding	33	1	0.27	0.00080
GO:0005548	phospholipid transporter activity	29	0	0.24	0.00185
GO:0003743	translation initiation factor activity	27	0	0.22	0.00303
GO:0016814	hydrolase activity, acting on carbon-nitrogen (but not peptide) bonds, in cyclic amidines	15	0	0.12	0.00324
GO:0099529	neurotransmitter receptor activity involved in regulation of postsynaptic membrane potential	25	2	0.21	0.00328
GO:0016538	cyclin-dependent protein serine/threonine kinase regulator activity	16	0	0.13	0.00616
GO:0140359	ABC-type transporter activity	24	0	0.20	0.00690
GO:0019239	deaminase activity	16	0	0.13	0.00977
GO:0140303	intramembrane lipid transporter activity	26	0	0.21	0.00987

Table S12: Molecular function GO term enrichment (weight01 algorithm and KS statistic) for PC2.

GO.ID	GO Term	Annotated	Significant	Expected	P-val
GO:0004896	cytokine receptor activity	32	0	0.21	0.0034
GO:0008201	heparin binding	40	1	0.26	0.0046
GO:0019239	deaminase activity	16	1	0.10	0.0048
GO:0051087	chaperone binding	50	1	0.32	0.0065
GO:0016405	CoA-ligase activity	13	1	0.08	0.0068
GO:0016298	lipase activity	51	0	0.33	0.0074
GO:0015318	inorganic molecular entity transmembrane transporter activity	372	1	2.39	0.0087
GO:0051082	unfolded protein binding	33	0	0.21	0.0094

Table S13: Molecular function GO term enrichment (weight01 algorithm and KS statistic) for PC3.

GO.ID	GO Term	Annotated	Significant	Expected	P-val
GO:0005212	structural constituent of eye lens	10	0	0.07	0.00036
GO:0140359	ABC-type transporter activity	24	0	0.18	0.00102
GO:0046961	proton-transporting ATPase activity, rotational mechanism	15	0	0.11	0.00149
GO:0003724	RNA helicase activity	32	0	0.24	0.00185
GO:0003743	translation initiation factor activity	27	0	0.20	0.00227
GO:0050660	flavin adenine dinucleotide binding	55	1	0.41	0.00344
GO:0051087	chaperone binding	50	2	0.37	0.00367
GO:0016791	phosphatase activity	161	3	1.20	0.00384
GO:0005524	ATP binding	741	9	5.53	0.00542
GO:0005104	fibroblast growth factor receptor binding	16	0	0.12	0.00560
GO:0004896	cytokine receptor activity	32	0	0.24	0.00635
GO:0008083	growth factor activity	56	0	0.42	0.00680
GO:0001664	G protein-coupled receptor binding	69	1	0.52	0.00716
GO:0008187	poly-pyrimidine tract binding	13	0	0.10	0.00892

Table S14: Molecular function GO term enrichment (weight01 algorithm and KS statistic) for PC4.

GO.ID	GO Term	Annotated	Significant	Expected	P-val
GO:0008017	microtubule binding	119	1	0.97	0.00068
GO:0016776	phosphotransferase activity, phosphate group as acceptor	18	0	0.15	0.00162
GO:0016831	carboxy-lyase activity	18	1	0.15	0.00425

Table S15: Cellular component GO term enrichment (weight01 algorithm and KS statistic) for PC1.

GO.ID	GO Term	Annotated	Significant	Expected	P-val
GO:0000307	cyclin-dependent protein kinase holoenzyme complex	24	0	0.19	0.00072
GO:0030496	midbody	98	2	0.76	0.00227
GO:0031234	extrinsic component of cytoplasmic side of plasma membrane	27	0	0.21	0.00292
GO:0000791	euchromatin	24	0	0.19	0.00540
GO:0008023	transcription elongation factor complex	26	0	0.20	0.00631
GO:0098797	plasma membrane protein complex	243	0	1.88	0.00638
GO:0080008	Cul4-RING E3 ubiquitin ligase complex	20	0	0.15	0.00697
GO:1902494	catalytic complex	738	8	5.71	0.00753
GO:0008076	voltage-gated potassium channel complex	50	0	0.39	0.00853
GO:0043209	myelin sheath	16	0	0.12	0.00876
GO:0043596	nuclear replication fork	18	0	0.14	0.00881

Table S16: Cellular component GO term enrichment (weight01 algorithm and KS statistic) for PC2.

GO.ID	Term	Annotated	Significant	Expected	P-val
GO:0008076	voltage-gated potassium channel complex	50	0	0.36	0.0011
GO:0016469	proton-transporting two-sector ATPase complex	22	0	0.16	0.0020
GO:0005576	extracellular region	501	2	3.61	0.0027
GO:0080008	Cul4-RING E3 ubiquitin ligase complex	20	0	0.14	0.0035
GO:0031462	Cul2-RING ubiquitin ligase complex	14	0	0.10	0.0035
GO:0005875	microtubule associated complex	43	0	0.31	0.0057
GO:0005743	mitochondrial inner membrane	109	2	0.78	0.0062
GO:0008180	COP9 signalosome	21	0	0.15	0.0070
GO:1902493	acetyltransferase complex	55	0	0.40	0.0082
GO:0031982	vesicle	723	4	5.20	0.0095
GO:0071944	cell periphery	1908	12	13.73	0.0096

Table S17: Cellular component GO term enrichment (weight01 algorithm and KS statistic) for PC3.

GO.ID	Term	Annotated	Significant	Expected	P-val
GO:0098797	plasma membrane protein complex	243	5	1.72	0.00053
GO:0030131	clathrin adaptor complex	10	0	0.07	0.00066
GO:1902493	acetyltransferase complex	55	1	0.39	0.00219
GO:0030684	preribosome	14	0	0.10	0.00430
GO:1990752	microtubule end	11	0	0.08	0.00677
GO:0016469	proton-transporting two-sector ATPase complex	22	0	0.16	0.00802

Table S18: Cellular component GO term enrichment (weight01 algorithm and KS statistic) for PC4.

GO.ID	Term	Annotated	Significant	Expected	P-val
GO:0005876	spindle microtubule	43	1	0.33	0.0011
GO:0032991	protein-containing complex	2429	25	18.47	0.0015
GO:1902494	catalytic complex	738	9	5.61	0.0023
GO:0000793	condensed chromosome	110	3	0.84	0.0038
GO:0098590	plasma membrane region	421	4	3.20	0.0073
GO:0043596	nuclear replication fork	18	0	0.14	0.0089

Table S19: Genes from significant GO term pathways associated with climate (this study) that overlap with heat-stress-associated genes in chickens: ADCY1, CACNA1C, CAMK2D, PACRG, PARK2, PRKCH, SDHD, SIRT1, WNT7B, TBXAS1, IL18, and VPS13C (Tian et al. 2020).

Trait	Gene	Role	GT_gene/pathway
Circulatory	ADCY1	Associated with adenylyl cyclase, affecting formation of cAMP, with roles in 4 pathways: vascular smooth muscle contraction, adrenergic singling in cardiomyocytes, melanogenesis, calcium singling pathway	Gene ADCY1 in GO:0065008 (PC1), GO:0048167 (PC2, PC4), GO:0007613 (PC2), GO:0006355 (PC3)
Mitochondrial respiration	VPS13C	Associated with maintenance of mitochondrial respiration (hypothesis role in Tian et al. 2020)	Gene VPS13C in GO:0010506 (PC4)
Kidney water reabsorption	DRD3	Related to Arachidonic acid secretion (GO:0050482) in Tian et al. 2020	DRD3 in the following: GO:0065008 (PC1), GO:0032467 (PC1), GO:0051726 (PC2), GO:0007186 (PC4), GO:0048878 (PC4)
Convergent adaptation between birds and mammals	TNFRSF11A	Tumor Necrosis Factor Receptor Superfamily. Association with Arachidonic acid metabolism in Tian et al. 2020	gene TNFRSF11A found in GO:0031325 (PC1), GO:0048534 (PC1), GO:0065008 (PC1), GO:0006355 (PC3), GO:0045859 (PC3)

Table S19: Twelve identified chicken heat stress genes by Tian et al. 2020 (*Continued*).

Trait	Gene	Role	GT_gene/pathway
Internal homeostasis	CACNA1C	voltage-dependent calcium channels	gene CACNA1C found in GO:0007507 (PC1), GO:0065008 (PC1), GO:0002027 (PC1), GO:0048878 (PC4)
Heat loss through blood vessels	PRKCH	vasoconstrictors	gene PRKCH found in GO:0006355 (PC3), GO:0008544 (PC3), GO:0010720 (PC3)

Table S20: Climate adaptation candidate genes from other studies that are closely related to candidate genes found here.

Species	Gene	Covariate	Species	Gene	Covariate	Ref	Biological Process
Parus major	ADAMTS2 (PCIMP)	PC4	Mandarin vole	ADAMTS2	Subterranean	Dong et al. 2018	downregulated in hypoxia response, perhaps to supply oxygen for cell survival
Parus major	BTBD7	PC3	Drosophila	BtbVii	isotherm; temp seasonality	Bogaerts-Márquez et al. 2021	protein ubiquitination by BTB
Parus major	BTBD7	PC3	Cattle	BTBD8	annual mean temp	Flori et al. 2019	protein ubiquitination by BTB
Chicken annotation of identified variants from current study	PDE1A	PC4	Drosophila	pde1c	max wind; mean diurnal range	Bogaerts-Márquez et al. 2021	Calmodium binding
Parus major	SLC25A42	PC4	Cattle	SLC46A1	PC2	Flori et al. 2019	solute carrier family

Table S20: Candidate closely related climate adaptation genes (*Continued*).

Species	Gene	Covariate	Species	Gene	Covariate	Ref	Biological Process
Parus major	TOX2	PC2	Cattle	TOX4	annual mean temp	Flori et al. 2019	transcription factor Tox high mobility group box family, role in T cell immunity
Parus major	KCNQ1	PC4	Cattle	KCNH1	PC2, temp annual range; annual precipitation; annual mean moisture index	Flori et al. 2019	Potassium voltage-gated channel subfamily genes, KCNQ1 required for repolarisation phase of the cardiac action potential.
Parus major	ZNF407	PC1	Cattle	ZNF445	temperature humidity index	Flori et al. 2019	Zinc finger protein, Zinc ion binding, DNA binding, transcriptional regulator
Parus major	dnajc10 (HSP40)	PC4	Cattle	HSPB3 (small heat shock protein)	climate	Flori et al. 2019	heat shock response

Table S20: Candidate closely related climate adaptation genes (*Continued*).

Species	Gene	Covariate	Species	Gene	Covariate	Ref	Biological Process
Parus major	CALM2	PC3, PC4	threespine sticklebacks	CALM1B	local environmental adaptation	Garcia-Elfring et al. 2021	Calcium transport
Parus major	ADAMTS2 (PCIMP)	PC4	Mandarin vole	ADAMTS2	Subterranean	Dong et al. 2018	downregulated in hypoxia response, perhaps to supply oxygen for cell survival

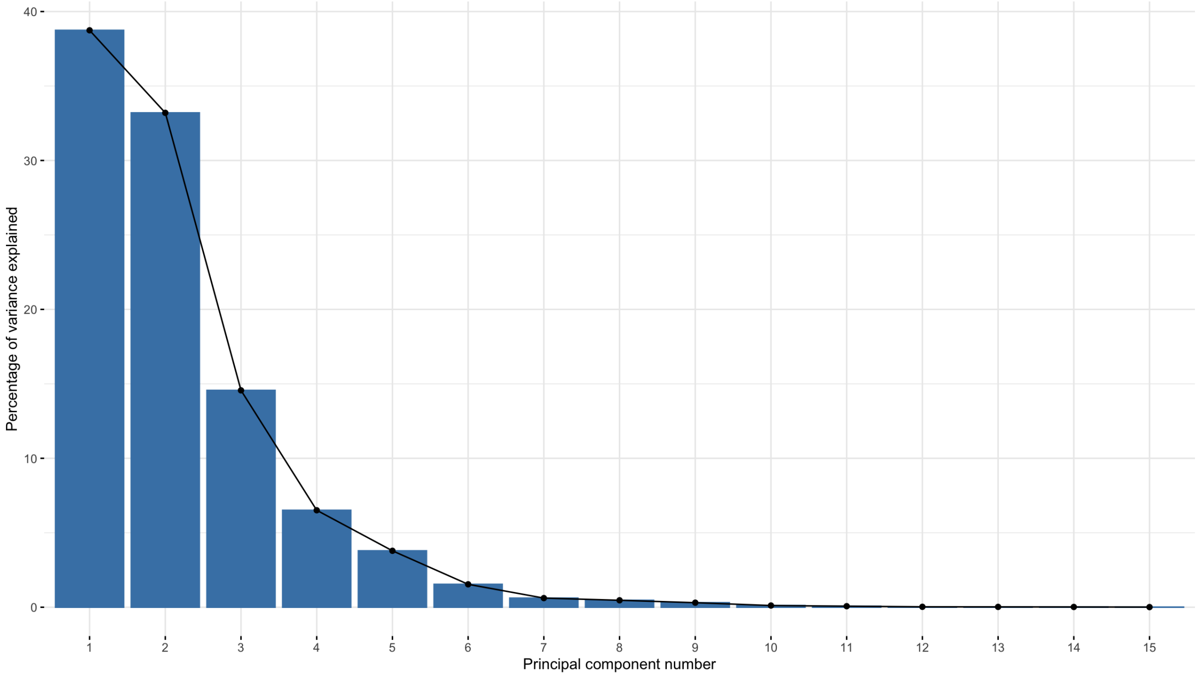

Figure S1: Scree plot summarising the principal components analysis of the 22 climate variables.

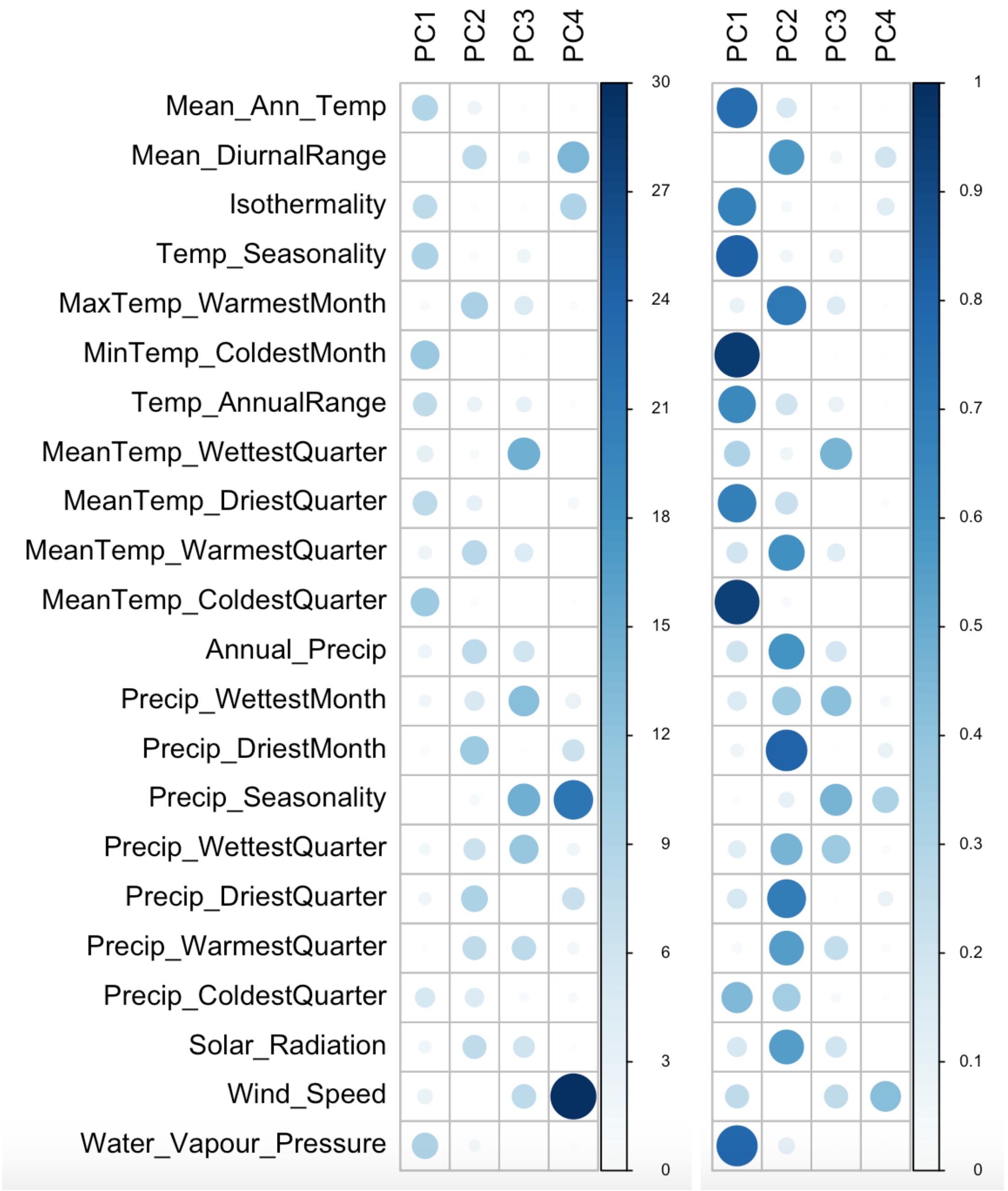

Figure S2: For the first four PCs: A) The contributions of climate variables to PCs (expressed as a percentage) are highlighted. B) The quality of representation of the climate variable on the PCA factor map plot is indicated by high cos2 values. The sum of cos2 values across all PCs of each variable equals one.

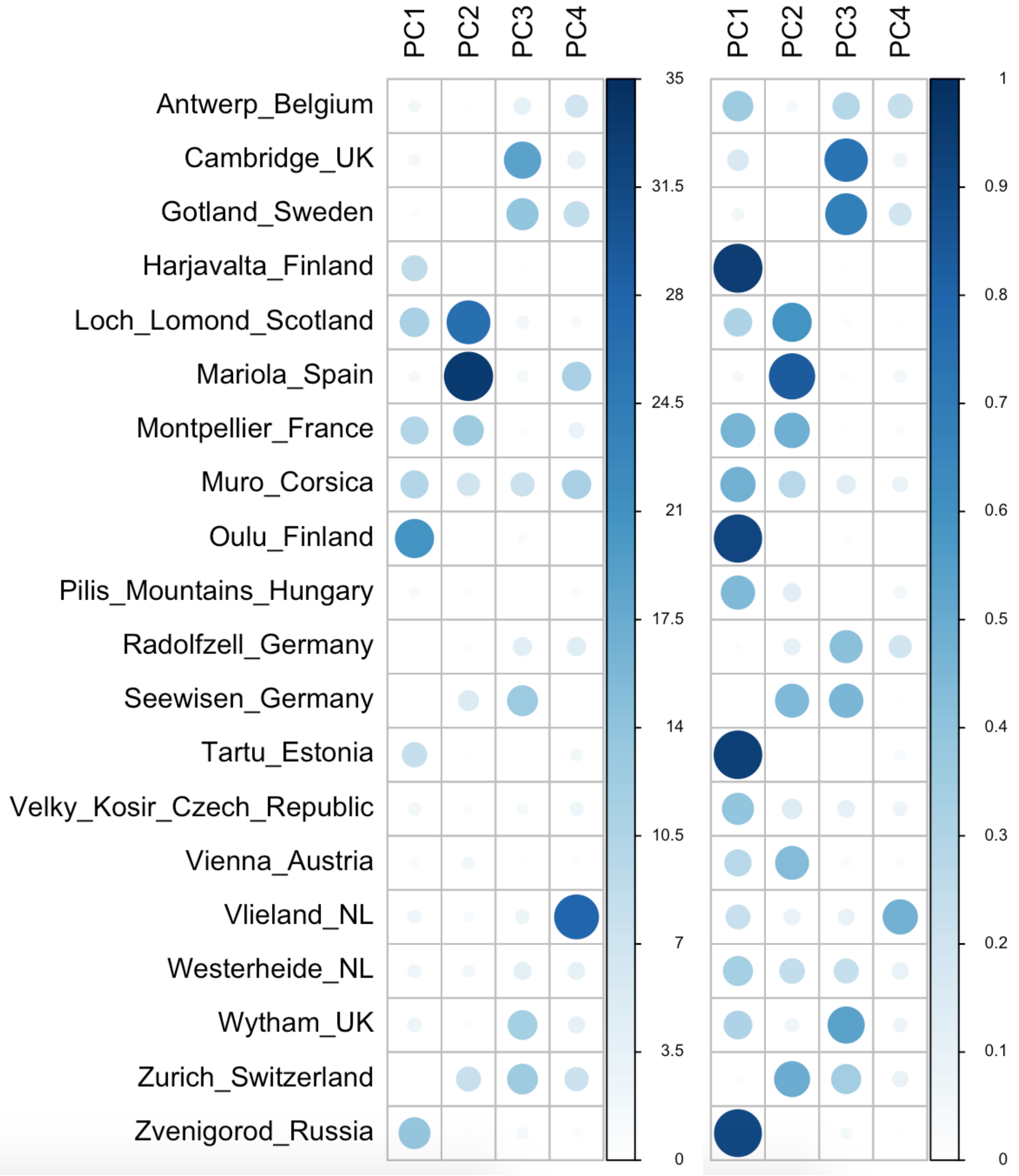

Figure S3: For the first four PCs: A) The contributions of populations to PCs (expressed as a percentage) are highlighted. B) The quality of representation of the population on the PCA factor map plot is indicated by high cos2 values. The sum of cos2 values across all PCs of each population equals one.

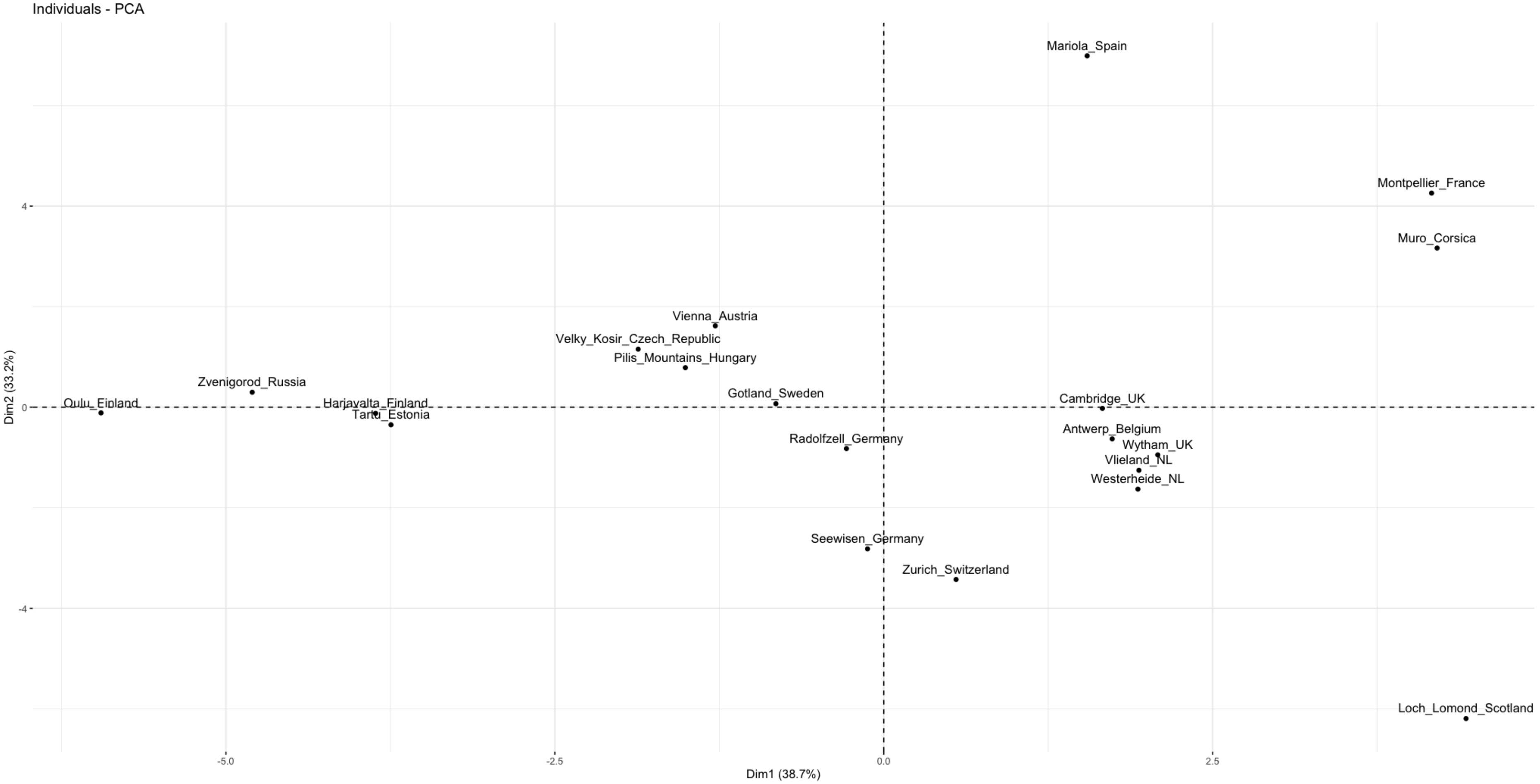

Figure S4: Scatterplot of the first two PCs of population climatic covariables

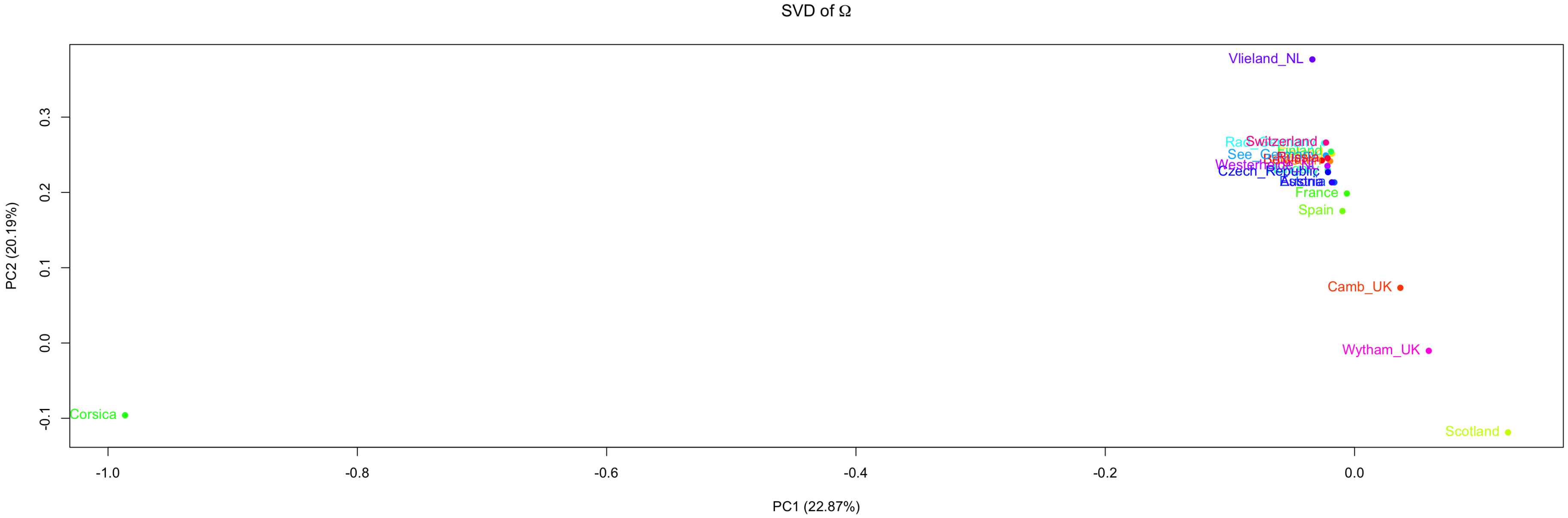

Figure S5: The first pair of Eigenvectors are visualised, produced from the scaled covariance matrix Omega of the population allele frequencies.

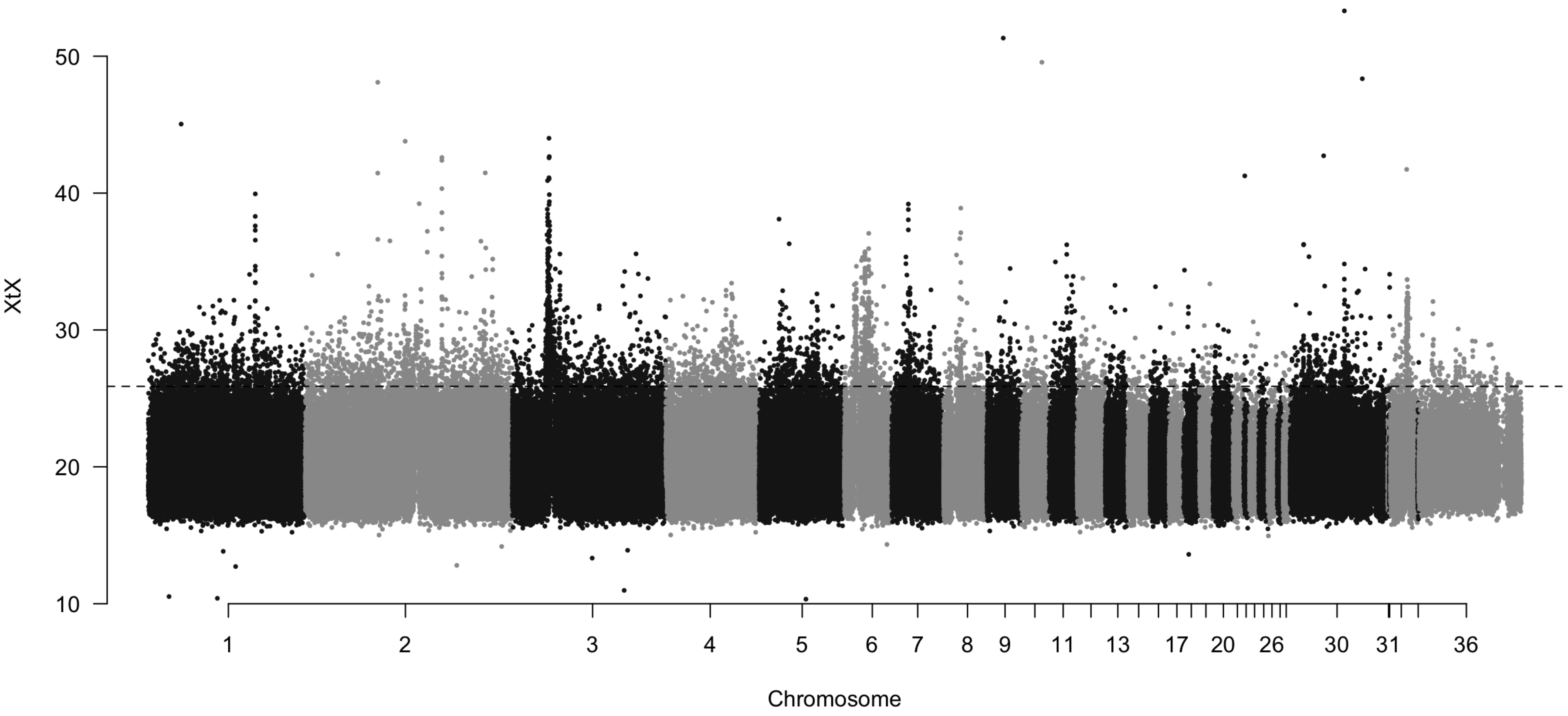

Figure S6: The genome-wide XtX statistic for each annotated variant, generated from Run 1 of the core model is plotted. The 1% significance threshold (XtXPOD 400,000) is indicated by the dashed line. SNPs found above the threshold are regarded as outlier loci, potentially under selection. Chromosomes 1-15, 17-24, 26-28, 30("1A"), 31, 32, 33("4A"), 34 and 36("Z") are displayed.

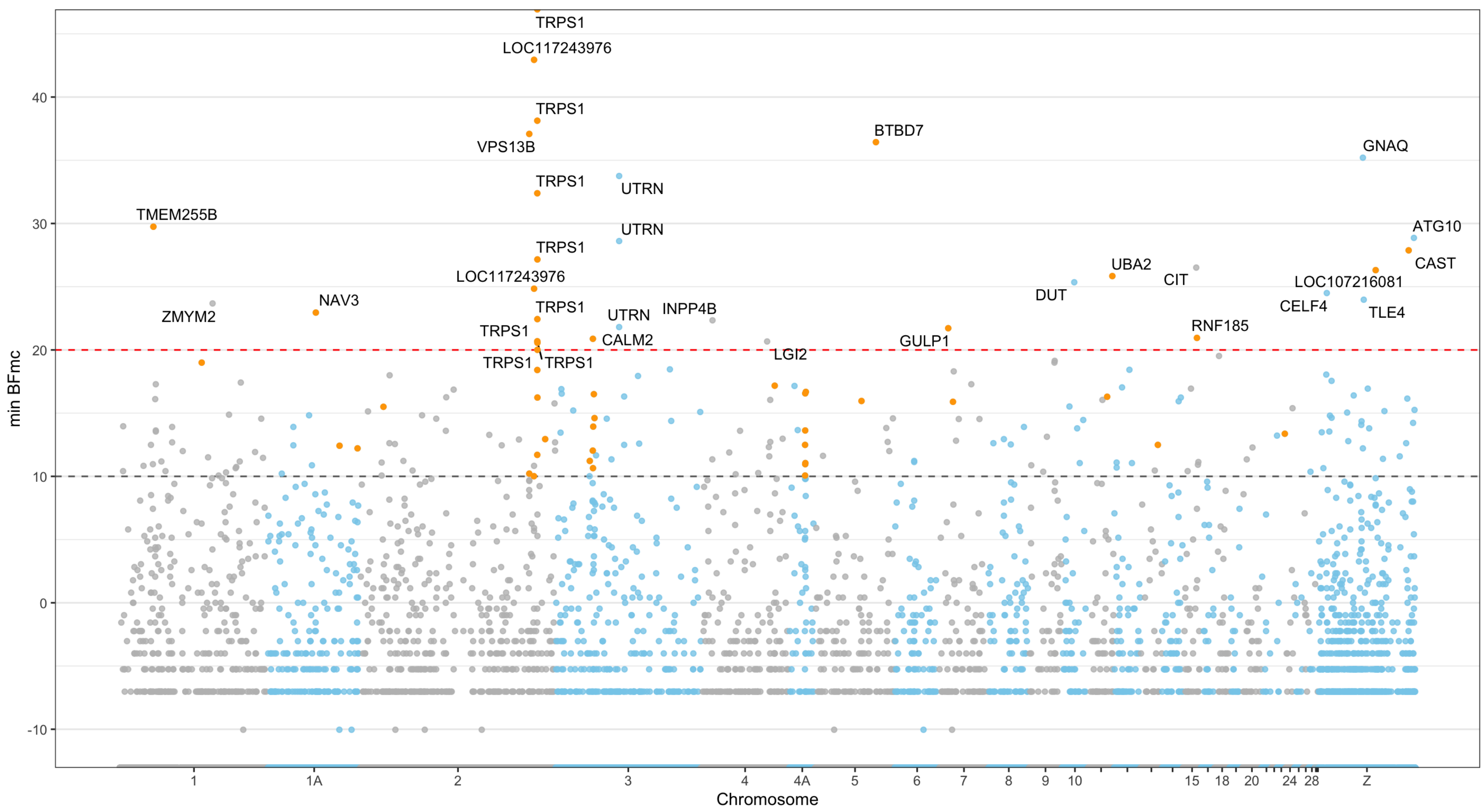

Figure S7: Manhattan plot of annotated genes associated with climate adaptation for PC3 at the decisive evidence threshold (BFmc>20). For all annotated variants, the minimum BFmc score across the 3 runs is shown. The strong (BFmc>10), very strong (BFmc>15) and decisive evidence thresholds are indicated by dashed lines. SNPs highlighted orange (at threshold BFmc>10) were also found to be under selection in the outlier locus analyses that did not consider climatic data. Chromosomes 1, 1A, 2-4, 4A, 5-15, 17-24, 26-28, 31-32, 34 and Z are displayed.

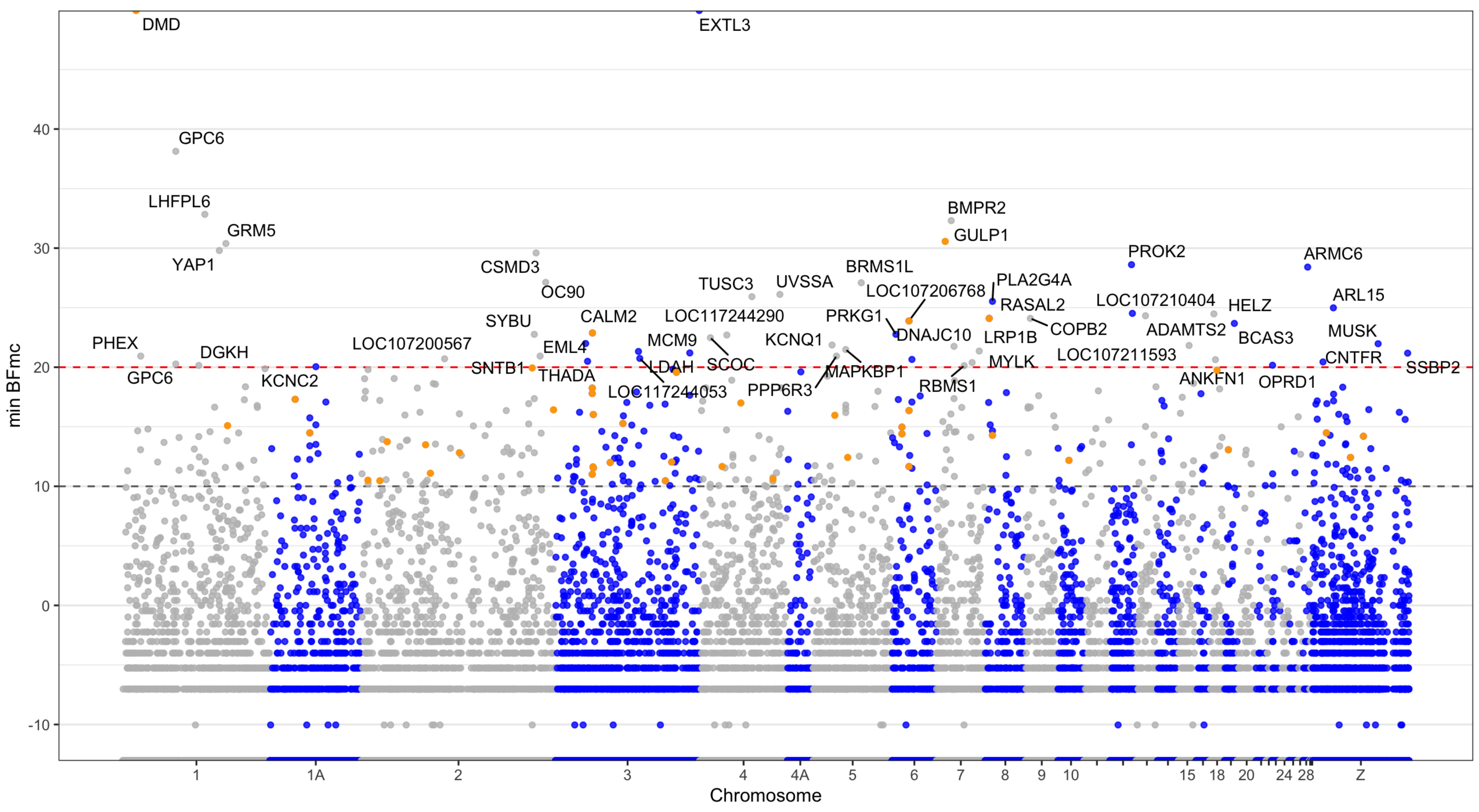

Figure S8: Manhattan plot of annotated genes associated with climate adaptation for PC4. The threshold of the decisive level of evidence (BFmc>20) is indicated along the plot, with annotated genes displayed for BFmc>20. Details as for Figure S7.

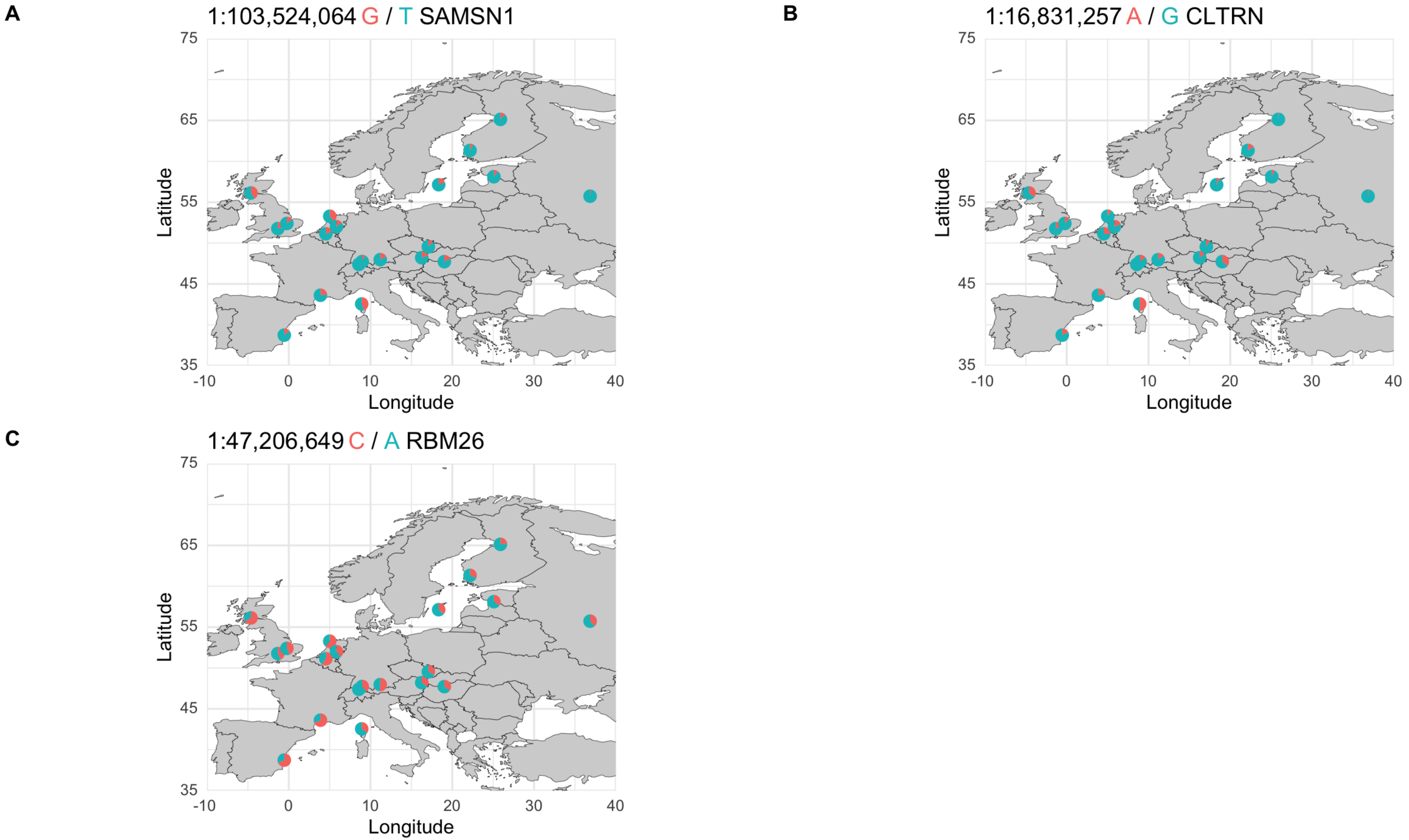

Figure S9: Allele frequencies in each population at loci associated with PC1 of climate variation (panels A-E). The title of each panel indicates the chromosome, position (bp), alleles and closest gene to each climate-associated SNP. Gene CLTRN is also termed TMEM27 in the chicken gene annotation.

D

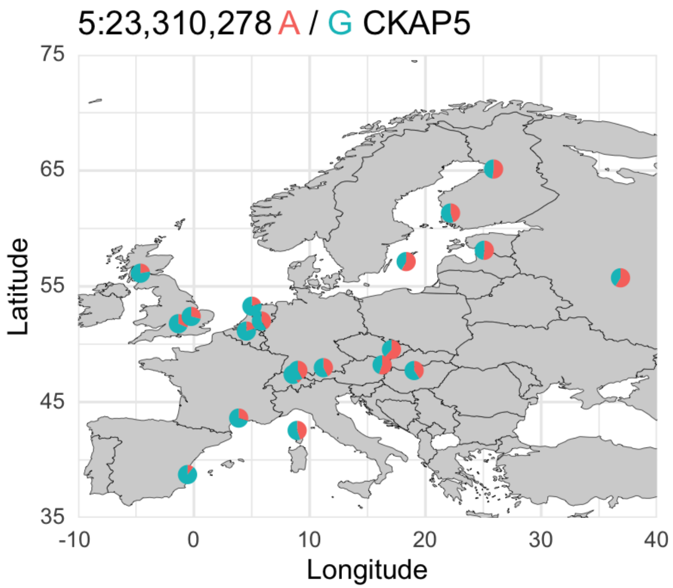

5:23,310,278 A / G CKAP5
Latitude
Longitude
75
65
55
45
35
-10
0
10
20
30
40

E

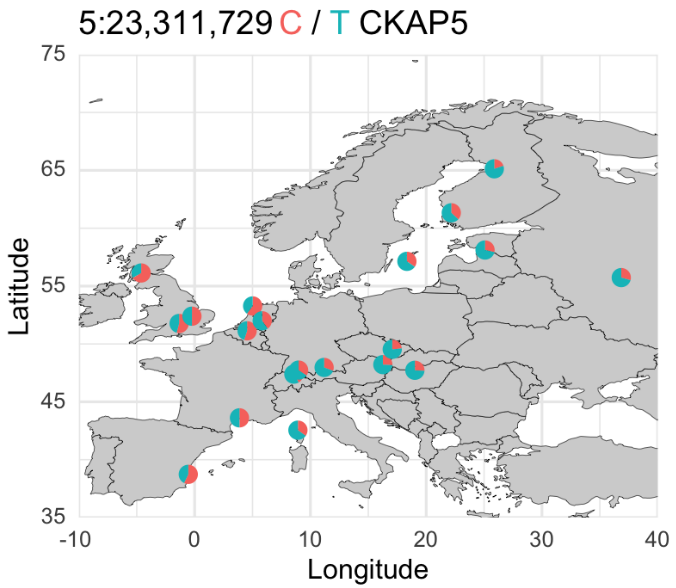

5:23,311,729 C / T CKAP5
Latitude
Longitude
75
65
55
45
35
-10
0
10
20
30
40

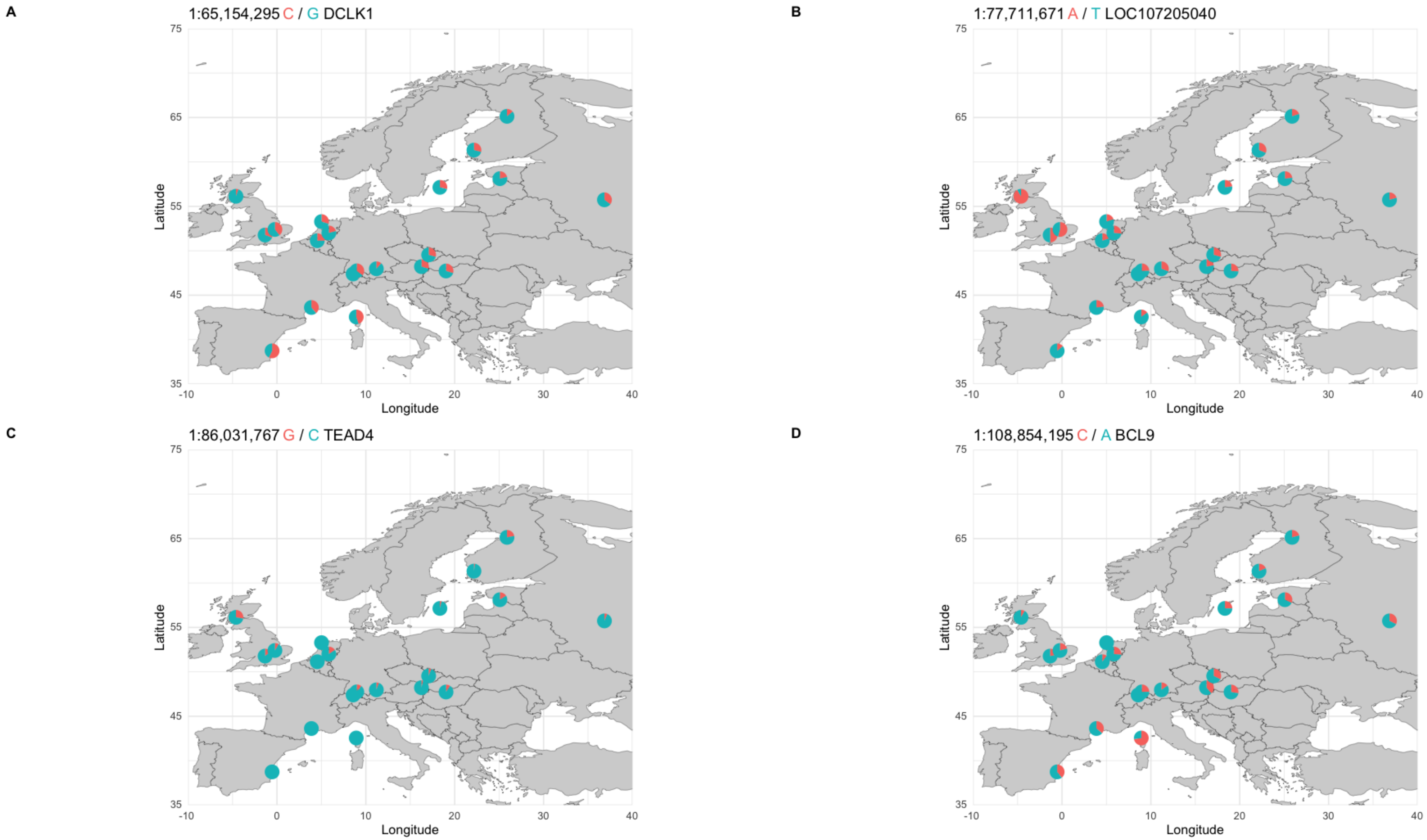

Figure S10: Allele frequencies in each population at loci associated with PC2 of climate variation (panels A-O). The title of each panel indicates the chromosome, position (bp), alleles and closest gene (annotated to Parus major genes) to each climate-associated SNP.

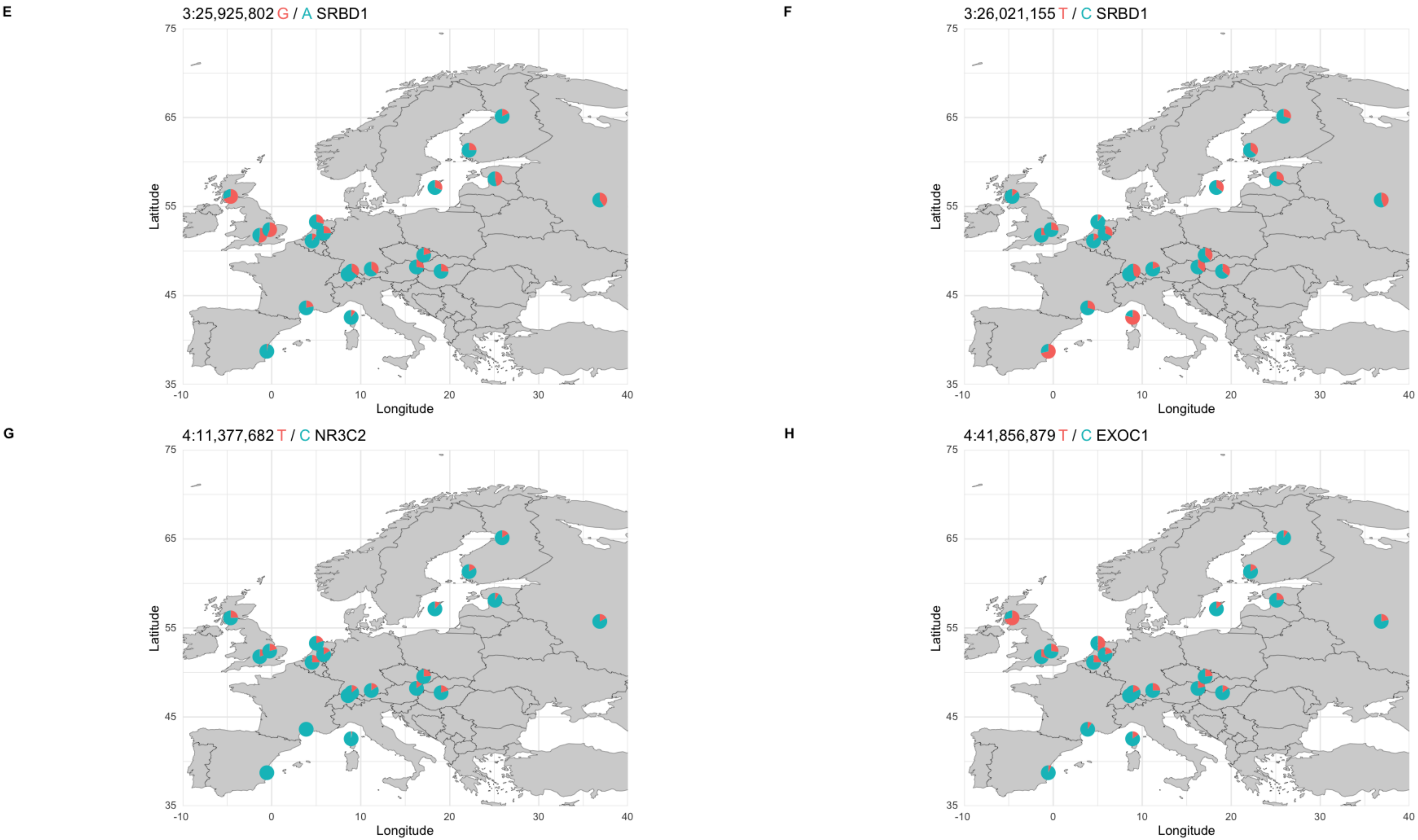
E
3:25,925,802 G / A SRBD1
F
3:26,021,155 T / C SRBD1
G
4:11,377,682 T / C NR3C2
H
4:41,856,879 T / C EXOC1
Latitude
Longitude
75
65
55
45
35
-10
0
10
20
30
40

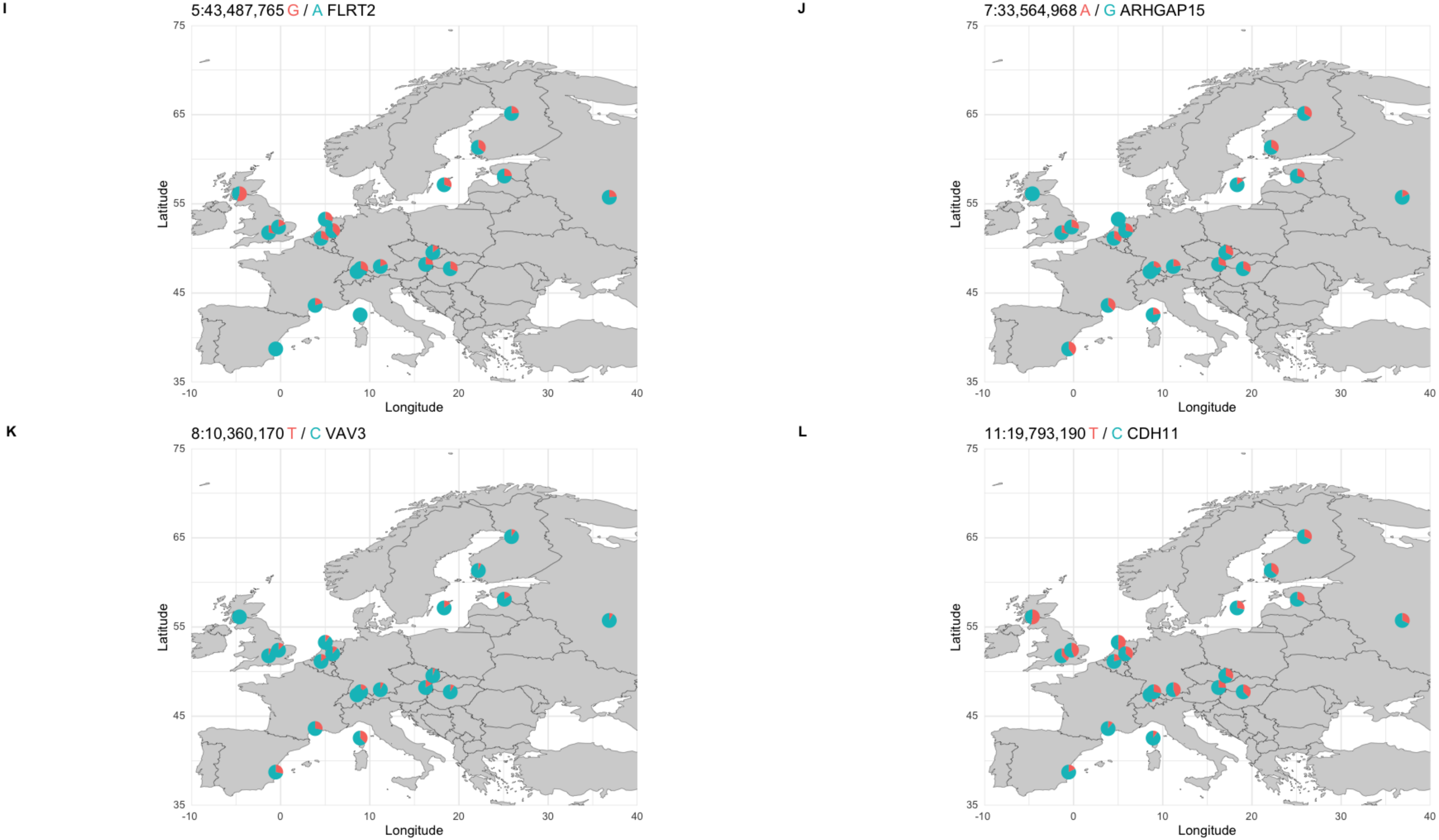

I
5:43,487,765 G / A FLRT2
J
7:33,564,968 A / G ARHGAP15
K
8:10,360,170 T / C VAV3
L
11:19,793,190 T / C CDH11
Latitude
Longitude
75
65
55
45
35
-10
0
10
20
30
40

M

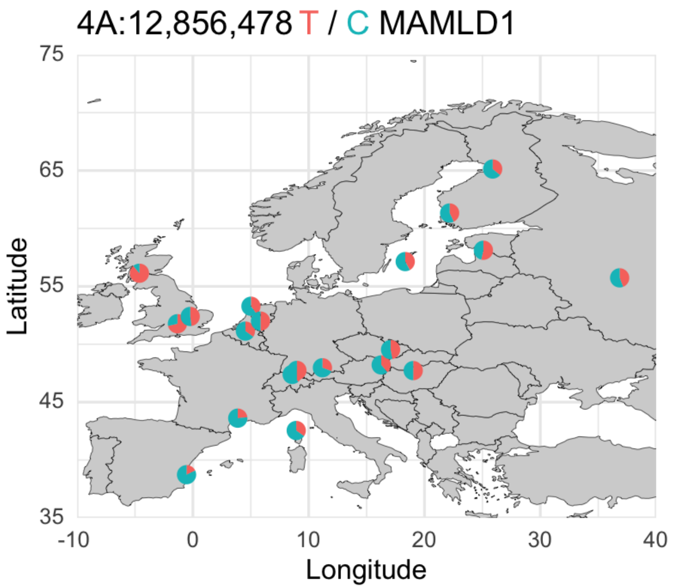
4A:12,856,478 T / C MAMLD1
Latitude
Longitude
75
65
55
45
35
-10
0
10
20
30
40

N

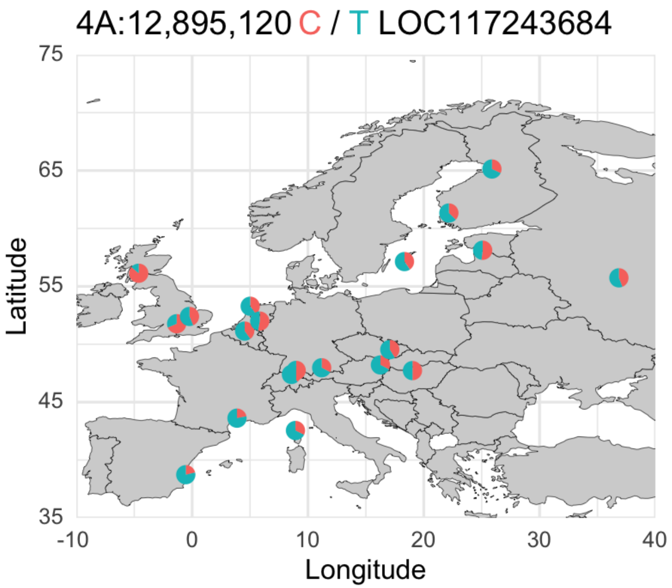
4A:12,895,120 C / T LOC117243684
Latitude
Longitude
75
65
55
45
35
-10
0
10
20
30
40

O

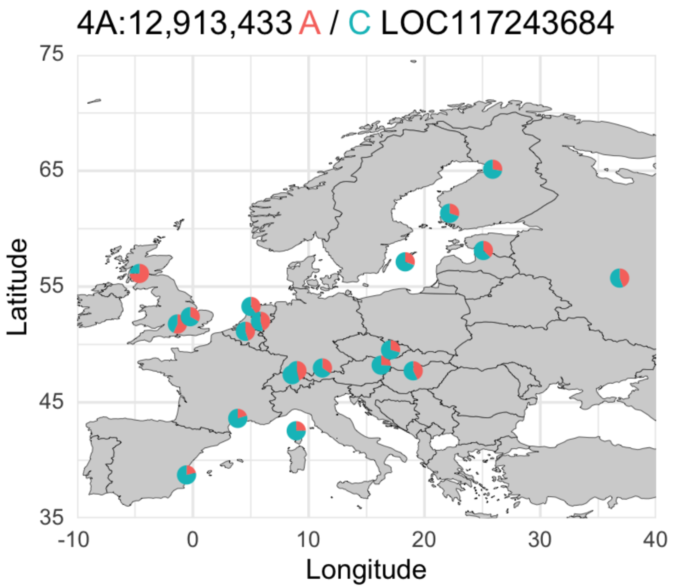
4A:12,913,433 A / C LOC117243684
Latitude
Longitude
75
65
55
45
35
-10
0
10
20
30
40

Additional references to main text: Table S20: Dong et al. (2018) and Garcia-Elfring et al. (2021).

#References

Dong, Qianqian, Luye Shi, Yangwei Li, Mengwan Jiang, Hong Sun, Baishi Wang, Han Cheng, et al. 2018. "Differential responses of Lasiopodomys mandarinus and Lasiopodomys brandtii to chronic hypoxia: A cross-species brain transcriptome analysis." *BMC Genomics* 19 (1): 1–11. https://doi.org/10.1186/s12864-018-5318-1.

Garcia-Elfring, Alan, Antoine Paccard, Timothy J. Thurman, Ben A. Wasserman, Eric P. Palkovacs, Andrew P. Hendry, and Rowan D. H. Barrett. 2021. "Using seasonal genomic changes to understand historical adaptation to new environments: Parallel selection on stickleback in highly-variable estuaries." https://doi.org/10.1111/mec.15879.
